# Heterologous Expression of Type II PKS Gene Cluster Leads to Diversified Angucyclines in *Streptomyces albus* J1074

**DOI:** 10.3390/md22110480

**Published:** 2024-10-22

**Authors:** Xiaoting Zhang, Falei Zhang, Chen Li, Jiayi Li, Xiao Xu, Tianjiao Zhu, Qian Che, Deihai Li, Guojian Zhang

**Affiliations:** 1 Key Laboratory of Marine Drugs, Chinese Ministry of Education, School of Medicine and Pharmacy, Ocean University of China, Qingdao 266003, China; 2 Laboratory for Marine Drugs and Bioproducts, Qingdao Marine Science and Technology Center, Qingdao 266237, China; 3 Sanya Oceanographic Institute, Ocean University of China, Sanya 572025, China; 4 Marine Biomedical Research Institute of Qingdao, Qingdao 266101, China

**Keywords:** heterologous expression, II PKS, marine *Streptomyces*, angucycline

## Abstract

Heterologous expression has emerged as an effective strategy in activating *Streptomyces* cryptic gene clusters or improving yield. Eight compounds were successfully obtained by heterologous expression of the type II PKS gene cluster *spi* derived from marine *Streptomyces* sp. HDN155000 in the chassis host *Streptomyces albus* J1074. The structures with absolute configurations were elucidated using extensive MS and NMR spectroscopic methods, as well as theoretical NMR calculations and electronic circular dichroism (ECD) calculations. Interestingly, compound WS009 Z (**2**) contains a rare thiomethyl group, angumycinone T (**4**) has a novel oxo-bridge formed between C12a and C4, and angumycinone X (**3**) showed cytotoxicity toward K562 and NCI-H446/EP cell lines.

## 1. Introduction

Angucyclines are a significant class of aromatic polyketide compounds biosynthesized by type II polyketide synthases (PKS) via an iterative Claisen condensation to generate Poly-β-ketone backbones [1]. So far, over 400 members of angucyclines have been reported [2] and nearly half of them showed a wide range of biological activities, such as antimicrobial and/or anticancer, glutamate receptor agonist, gastric mucosal protectant, and platelet aggregation-inhibiting actions [3], which continually enhanced the importance of expanding the chemical space and obtaining novel angucycline derivatives for drug discovery and development purposes [4].

*Streptomyces*, a type of actinobacteria, is a Gram-positive bacterium with high GC genomes, primarily isolated from soil and marine sediments and other habitat samples [5,6,7,8], also known for its ability to produce a complex variety of secondary metabolites [9,10,11]. As genome sequencing technology advances, an increasing number of gene clusters (BGC) for natural products are being discovered [12]. However, only a limited number of natural products have been identified to date [13], making it essential to convert genomic information into valuable compounds for drug discovery and also commercial production. Heterologous expression of gene clusters is an effective strategy to address this problem by enabling the activation of cryptic gene clusters in heterologous host strains and increasing the yield of gene clusters expressed at low levels under laboratory conditions, as well as obtaining new analogues other than wild-type [14,15,16]. However, the biosynthetic capacity of an exogenous gene cluster is greatly affected by the metabolic characteristics of the chassis cells; for example, the competitive consuming of precursors in the native biosynthetic pathways will stop or drastically decrease the production of the desired compound. For this reason, it is critical to select a proper surrogate host, because different hosts influence the yield and abundance of compounds, even possibly producing entirely distinct compounds [17,18]. Thus far, various *Streptomyces* species including *S. coelicolor*, *S. lividans*, *S. venezuelae*, *S. albus*, *S. fradiae*, *S. roseosporus*, and *S. toyocaensis* have been specifically engineered and frequently employed for heterologous expression [19].

During our ongoing research into bioactive chemicals from marine-derived microorganisms, six angucycline derivatives, including one novel oxaspirocyclic structure, were obtained by heterologous expression of a type II polyketide biosynthesis gene cluster *spi* in *Streptomyces coelicolor* A3(2) [20]. In the current study, to further explore the metabolic capacity of the BGC of *spi* and acquire more angucycline analogues, this cluster was expressed in another feasible heterologous host, *S. albus* J1074. A total of six angucycline derivatives and two by-products were obtained from the heterologous strains. Here, we will describe the construction of the recombinant strain, and the characterization of the structures obtained from the culture.

## 2. Results

### 2.1. Heterologous Expression of spi BGC in S. albus J1074

**Figure 1:**
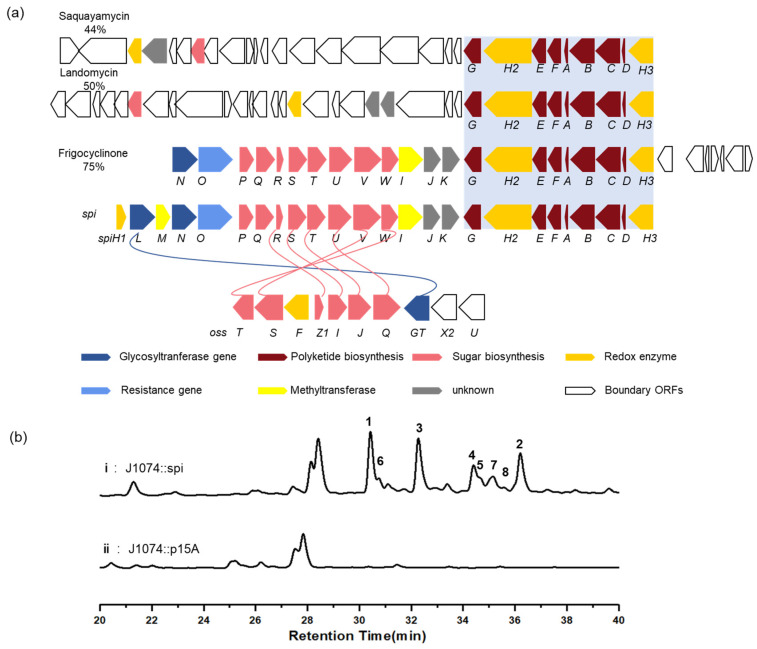
(**a**) The *spi* gene cluster in comparison to homologous gene clusters that encode saquayamycin, landomycin, frigocyclinone, and ossamine (*oss*). (**b**) ⅰ: HPLC analysis of heterologous expression strain J1074::spi; ⅱ: HPLC analysis of negative control host strain *S. albus* J1074::p15A.

A type II PKS gene cluster *spi* (GenBank accession number OP009365, Figure 1a), which is responsible for angucyclines synthesis, has been previously identified and confirmed from the genome of a marine-derived actinobacteria strain of *Streptomyces* sp. HDN155000 [20]. To develop the metabolic potential of the *spi* BGC and obtain more angucycline analogues, we decided to have *spi* expressed in *S. albus* J1074, which has been demonstrated as a friendly surrogate host for heterologously expressing actinobacteria sourced genes due to its faster growth, small genome size, easy genetic manipulation procedure, and clear metabolic background [21,22,23]. The empty vector p15A was transferred into *S. albus* J1074 as the negative control (Figure 1b, trace i), and a series of new peaks were detected by HPLC analysis (Figure 1b, trace ii), demonstrating that the *spi* gene cluster was successfully activated and that changing heterologous host would greatly develop the metabolic capacity of the *spi* BGC (Figure S2).

### 2.2. Isolation and Purification of Compounds from Recombinant Strain

**Figure 2:**
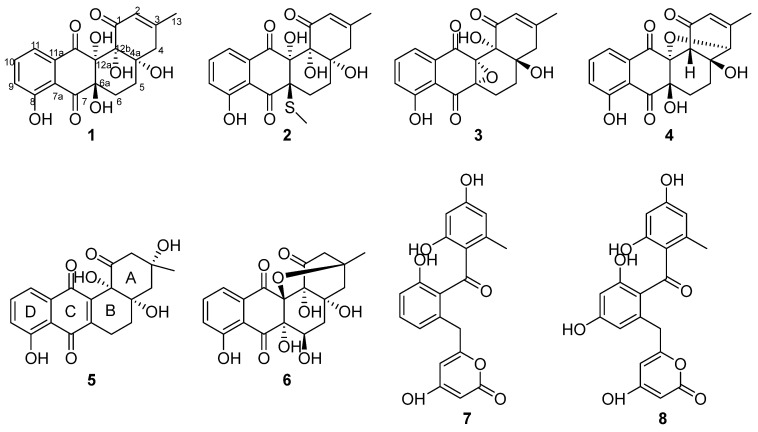
Chemical structures of **1**–**8**.

To specifically identify the compounds, we processed a larger-scale fermentation (20 L) of the strain J1074::spi, and ethyl acetate extraction of the fermentation product yielded 11.3 g crude extract. Subsequent stepwise separation by column chromatography (CC) with SiliaSphere C18 (ODS), medium pressure liquid chromatography (MPLC) and HPLC of the crude extract yielded a total of eight compounds, including four undescribed angucyclinone analogues: angumycinone Z (**1**) (7.1 mg), WS009 Z (**2**) (5.6 mg), angumycinone X (**3**) (6.4 mg), and angumycinone T (**4**) (4.7 mg). Compounds **5**–**8** were identified by comparing their 1D NMR and MS data to previously published literature (Figure 2).

**Figure 3:**
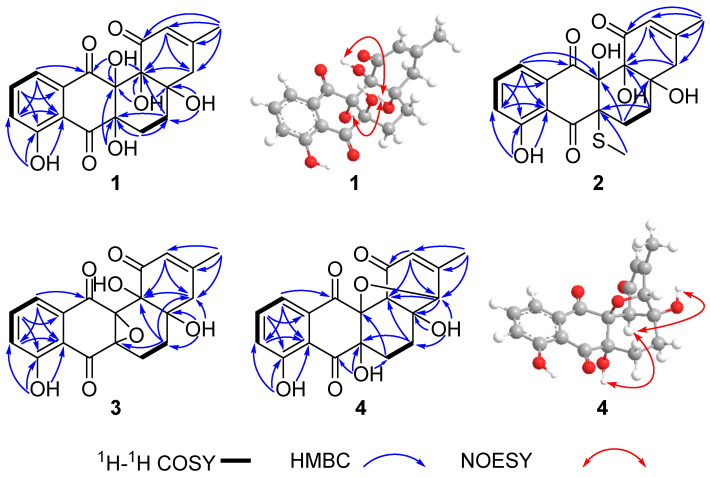
^1^H-^1^H COSY, key HMBC and NOESY correlations of **1**–**4**.

Compound **1** was separated as a pale brown powder. Its molecular formula, C_19_H_18_O_8_, was identified by HR-ESIMS *m*/*z* 373.0916 [M − H]^−^, indicating eleven degrees of unsaturation. The signals indicated in the ^1^H NMR data illustrate that there are one methyl (*δ*_H_ 1.93, s), three methylenes (*δ*_H_ 2.89, 2.06; *δ*_H_ 1.98, 1.60; *δ*_H_ 2.28, 1.74), one vinyl methine (*δ*_H_ 5.71, s), three aromatic methines (*δ*_H_ 7.34, d, *J* = 8.2 Hz; *δ*_H_ 7.76, t, *J* = 8.0 Hz; *δ*_H_ 7.60, d, *J* = 7.5 Hz), and five exchangeable protons (*δ*_H_ 11.10, s; *δ*_H_ 6.32, s; *δ*_H_ 6.03, s; *δ*_H_ 7.01, s; *δ*_H_ 5.66, s) (Table 1). The ^13^C NMR spectrum combined with the HSQC spectrum reveals the existence of one methyl, three sp3 methylenes, four protonated sp2 carbons, and eleven quaternary carbons (Table 2). These data imply that compound **1** has a tetracyclic benz[a]anthracene skeleton belonging to the angucycline analogue [24,25], which possibly resulted from the expression of the *spi* BGC. The planar structure of **1** was subsequently identified through a comprehensive analysis of 2D NMR data (Figure 3). Firstly, the ^1^H-^1^H COSY correlations from H-9 (*δ*_H_ 7.34, d, *J* = 8.2 Hz) via H-10 (*δ*_H_ 7.76, t, *J* = 8.0 Hz) through H-11 (*δ*_H_ 7.60, d, *J* = 7.5 Hz) indicate the presence of a 1,2,3-trisubstituted benzene ring (ring D) (Figure 3). The HMBC spectrum correlations from 8-OH (*δ*_H_ 11.10) to C-8 (*δ*_C_ 159.9, s), C-9 (*δ*_C_ 123.6, d), and C-7a (*δ*_C_ 116.3, s) showed a phenolic OH at C-8. The HMBC correlations from Me-13 to C-4 (*δ*_C_ 41.8, t), C-3 (*δ*_C_ 158.2, s), C-1 (*δ*_C_ 192.8, s), and C-2 (*δ*_C_ 122.9 d), from H-2 (*δ*_H_ 5.71, s) to C-4 and C-12b (*δ*_C_ 76.2, s), as well as from H-4 (*δ*_H_ 2.06, d, *J* = 17.7 Hz) to C-4a (*δ*_C_ 75.5, s) and C-12b (*δ*_C_ 76.2, s) identified the presence of the A ring. The B ring was determined via the HMBC correlations from the active exchangeable protons 12a-OH (*δ*_H_ 7.01) to C-12a (*δ*_C_ 76.4, s), C-12 (*δ*_C_ 194.1, s), and C-12b (*δ*_C_ 76.2, s), from 12b-OH (*δ*_H_ 5.66) to C-4a (*δ*_C_ 75.5, s), C-12a (*δ*_C_ 76.4, s), and C-12b, from 4a-OH (*δ*_H_ 6.32) to C-4a, and C-5 (*δ*_C_ 28.2, t), and from 6a-OH (*δ*_H_ 6.03) to C-6a (*δ*_C_ 77.6, s) and C-12a (*δ*_C_ 76.4, s). The HMBC correlations from H-11 to C-12 and C-7 (*δ*_C_ 200.5, s) confirmed the connection of rings B and D via ring C. Thus, the planar structure of **1** was identified (Figure 3).

**Figure 4:**
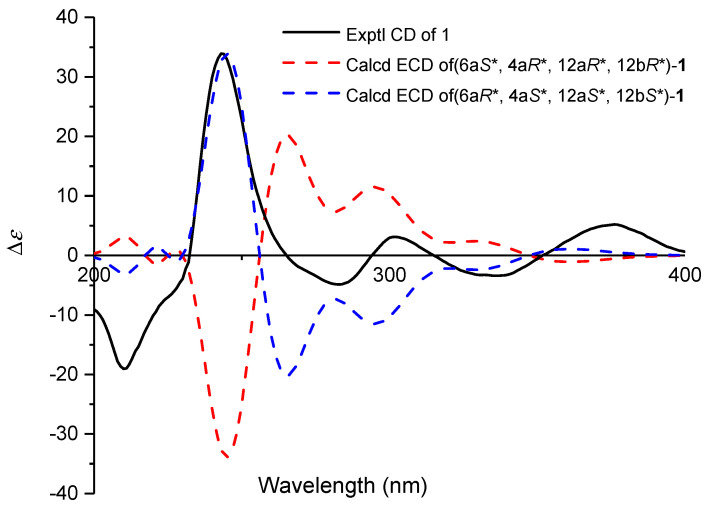
Calculated and experimental ECD spectra of **1**.

The absolute configuration of **1** was determined by NOESY spectrum analysis and ECD calculations. Analyzing the NOESY spectrum data revealed a correlation between 4a-OH (*δ*_H_ 6.32) and 12a-OH (*δ*_H_ 7.01), as well as a correlation between 4a-OH and 12b-OH (*δ*_H_ 5.66), while there was no correlation with 6a-OH (*δ*_H_ 6.03), which indicated that 4a-OH, 12a-OH, and 12b-OH were all in the same plane and 6a-OH was on the opposite side. Thus, the relative configuration of **1** was confirmed to be (6aS*, 4aR*, 12aR*, 12bR*)-**1** or (6aR*, 4aS*, 12aS*, 12bS*)-**1**. ECD calculation demonstrated that the latter was more compatible with the measured values, based on which the absolute configuration of compound **1** was confirmed as (6a*R*, 4a*S*, 12a*S*, 12b*S*)-**1** (Figure 4).

**Scheme 1:**
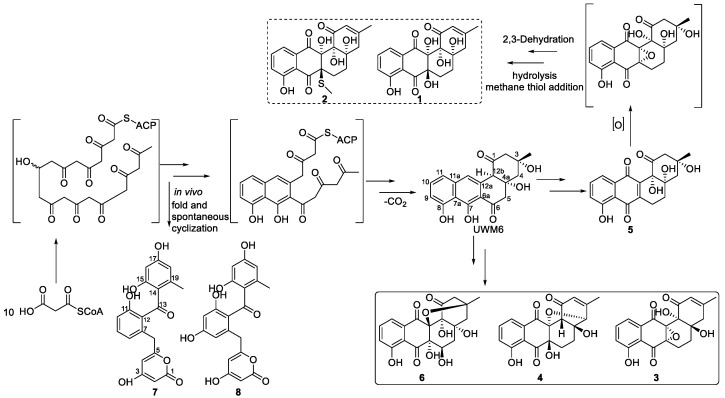
Proposed biosynthetic pathways of compounds **1**–**8**.

Compound **2** is an even brown amorphous powder with a molecular formula of C_20_H_20_O_7_S given based on HRESIMS data. One-dimensional NMR and HSQC spectra showed that there were two methyl (*δ*_C_ 23.7 and 13.3), three sp3 methylenes (*δ*_C_ 17.9, 28.8, and 41.6), four protonated sp2 carbons (*δ*_C_ 119.0, 122.7, 124.4, and 136.0), and eleven quaternary carbons including four non-protonated sp3 carbons (*δ*_C_ 61.2, 75.7, 75.7, and 78.4), four non-protonated sp2 carbons (*δ*_C_ 115.4, 131.5, 159.4, and 160.0), and three carbonyls (*δ*_C_ 191.0, 193.5, and 195.5) (Table 2). These signals all indicate that compound **2** has a backbone highly similar to **1**. The difference lies in the high-field chemical shift of C-6 (*δ*_C_ 17.9, t), C-6a (*δ*_C_ 61.2, s), and C-7 (*δ*_C_ 195.5, s), recombined with the HMBC correlation from -SCH_3_ (*δ*_H_ 1.67) to C-6a (Figure 3) and the HR-ESIMS data, which determined that the hydroxyl signal (6a-OH) in compound 1 was replaced by the thiomethyl group (δ_C_ 13.3, q) in **2**. However, the NOESY data could not determine the relative configuration of the thiomethyl group and three hydroxyl groups. Compounds 2 and 1 are proposed to have originated from the same epoxide precursor (discussed later in Scheme 1), which went through a ring opening process probably promoted by hydrolysis or methane thiol addition, hence the absolute configuration of **2** was tentatively assigned as 6a*R*, 4a*S*, 12a*S*, 12b*S*.

**Figure 5:**
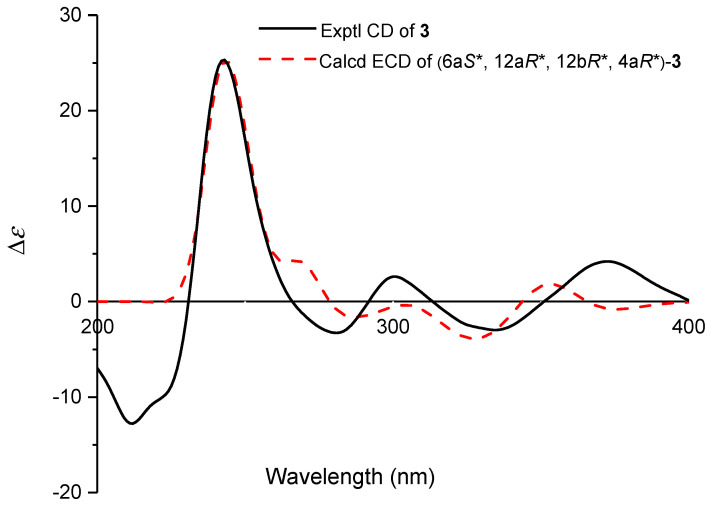
Calculated and experimental ECD spectra of **3**.

Compound **3** is a yellowish solid powder. HR-ESIMS analysis established the chemical formula as C_19_H_16_O_7_, with twelve degrees of unsaturation. The ^1^H and ^13^C NMR spectra revealed that **3** contains one methyl, three methylene, four methenyl, and 11 quaternary carbon signals (including three carbonyl and six aromatic quaternary carbons). The ^1^H and ^13^C NMR spectra showed that **3** was highly similar to angumycinone A [26], with the only difference being the low-field chemical shift at C-12a (*δ*_C_ 67.5), from which it can be deduced that there is hydroxylation at this position, thus determining the planar structure of compound **3**. Nevertheless, the relative configuration was unable to be defined by the NOESY data. The stereo-structure was determined based on NMR calculations, DP4+ analysis, and ECD calculations, primarily NMR calculations and DP4+ probability analyses of the four possible configurations (6aS*, 12aR*, 12bS*, 4aR*)-**3a**, (6aS*, 12aR*, 12bR*, 4aS*)-**3b**, (6aS*, 12aR*, 12bS*, 4aS*)-**3c**, and (6aS*, 12aR*, 12bR*, 4aR*)-**3d** at the B3LYP/6-311+G(d,p) level using PCM model in DMSO. The calculated results for **3d** (*R*^2^ = 0.9958) were more consistent with the experimental data than others (Figure S3). Furthermore, the DP4+ probability calculations identified **3d** with a 99.90% probability (Table S7). The absolute configuration of **3** was then determined to be 6a*S*,12a*R*,12b*R*,4a*R* through ECD calculation at the B3LYP/6-31+G(d,p)//B3LYP/6-31G(d) level was compared with the experimental ECD curve (Figure 5).

Compound **4** is a brown amorphous powder with molecular formula C_19_H_16_O_7_ deduced from HR-ESIMS, indicating 12 degrees of unsaturation. The ^1^H NMR spectrum of **4** displayed one methyl (*δ*_H_ 2.33, d, *J* = 1.2 Hz), two methylenes (*δ*_H_ 1.98; *δ*_H_ 2.24, 2.14), five methines (*δ*_H_ 3.98, d, *J* = 1.3 Hz; *δ*_H_ 3.14, s; *δ*_H_ 7.35, dd, *J* = 8.6, 1.0 Hz; *δ*_H_ 7.76, dd, *J* = 8.3, 7.6 Hz; *δ*_H_ 7.34, d, *J* = 7.5, 1.0 Hz), a vinyl methine (*δ*_H_ 5.83, s), and three exchangeable protons (*δ*_H_ 5.59, s; *δ*_H_ 7.05, s; *δ*_H_ 11.72, s) (Table 1). The ^13^C NMR and HSQC spectrum showed that **4** contains one methyl (*δ*_C_ 26.3, q), two methylene (*δ*_C_ 26.8 and 32.6), six methines (*δ*_C_ 52.7, 85.1, 118.3, 123.7, 124.6, and 137.7), and ten quaternary carbons, including three non-protonated sp3 carbons (*δ*_C_ 78.0, 81.6 and 92.5), four non-protonated sp2 carbons (*δ*_C_ 114.9, 135.3, 161.1, and 162.4), and three carbonyls (*δ*_C_ 192.1, 195.6, and 201.6) (Table 2). The planar structure of **4** was then identified via a detailed analysis of 2D NMR (Figure 3). The A ring was determined by the HMBC correlations from H-2 (*δ*_H_ 5.83, s) to C-1 (*δ*_C_ 195.6), C-4 (*δ*_C_ 85.1), and C-12b (*δ*_C_ 52.7), from 13-CH_3_ (*δ*_H_ 2.33, d) to C-2 (*δ*_C_ 124.6), C-3 (*δ*_C_ 162.4), C-4, and from H-4 (*δ*_H_ 3.98, d, *J* = 1.3 Hz) to C-4a (*δ*_C_ 81.6) and C-12b. The ^1^H-^1^H COSY correlations from H-9 (*δ*_H_ 7.35, dd, *J* = 8.6, 1.0 Hz) via H-10 (*δ*_H_ 7.76, dd, *J* = 8.3, 7.6 Hz) through H-11(*δ*_H_ 7.34, dd, *J* = 7.5, 1.0 Hz) suggested the presence of the 1,2,3-trisubstituted benzene ring (ring D). HMBC correlation analyses from H-9 to C-7a (*δ*_C_ 114.9) and C-7 (*δ*_C_ 201.6), from H-10 to C-11a (*δ*_C_ 135.3) and C-8 (*δ*_C_ 161.1), and from H-11 to C-12 (*δ*_C_ 192.1) extend ring D to ring C. Further analysis of the HMBC correlations from H-5 (*δ*_H_ 1.98, m) to C-6a (*δ*_C_ 78.0) and C-12b and from H-6 (*δ*_H_ 2.14) to C-12a (*δ*_C_ 92.5) determined the presence of ring B, closing the angular skeleton of compound **4**. HMBC correlations from the active exchangeable protons 4a-OH (*δ*_H_ 5.59) to C-4a, C-4 and C-5 (*δ*_C_ 32.6), from 6a-OH (*δ*_H_ 7.05, s) to C-6a, C-7 and C-6, together with the fact that there are 12 degrees of unsaturation, suggests that another unsaturation should be the presence of an epoxide between C-12a and C-4 (*δ*_C_ 85.1). Thus, the planar structure of **4** was determined.

**Figure 6:**
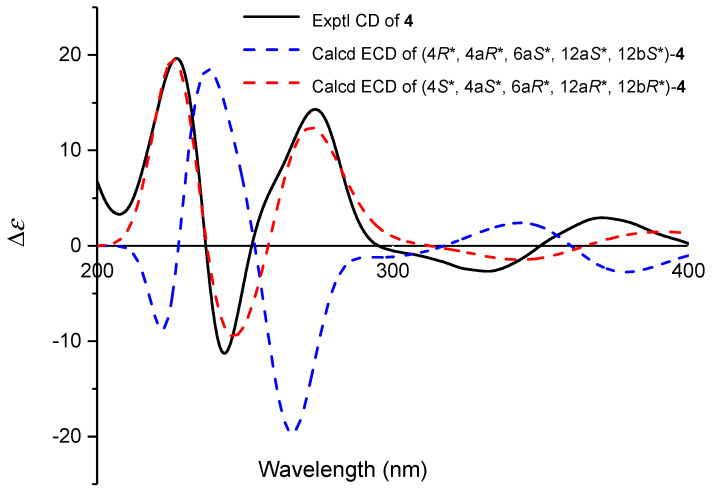
Calculated and experimental ECD spectra of **4**.

The stereo configuration of compound **4** was determined by NOESY spectral analysis and ECD calculations. The NOESY spectrum analysis revealed that H-12b (*δ*_H_ 3.14) correlates with 4a-OH and 6a-OH, indicating that H-12b, 4a-OH, and 6a-OH are in the same plane, resulting in only two possible structures: (4R*, 4aR*, 6aS*, 12aS*, 12bS*)-**4** and (4S*, 4aS*, 6aR*, 12aR*, 12bR*)-**4**. Finally, the absolute configuration of **4** was determined to be 4*S*,4a*S*,6a*R*,12a*R* based on the results of ECD calculations (Figure 6). Angucyclines carrying epoxide modifications on the skeletons were frequently discovered in nature [27], however, this is the first example of angucycline derivative with the epoxidation-bridged C-12a and C-4.

Based on the comparison of NMR and MS data with those reported in the literature, the six known compounds isolated in this study were identified as WP 3688-2 (**5**) [28], gephyromycin (**6**) [29,30], SEK43 (**7**) [31], and SEK15 (**8**) [32].

All new angucyclines were evaluated for cytotoxicity against L-02, MDA-MB-231, K562, ASPC-1, and NCI-H446/EP cell lines in vitro. Adriamycin was used as the positive control. Compound **3** showed cytotoxicity toward five cancer cell lines and especially strongly inhibited K562 and NCI-H446/EP cell lines with IC50 values of 11.72 and 8.92 μM, respectively. It is worth mentioning that both compounds **3** and **4** possess an oxygen bridge and two hydroxyl groups in the structure; however, compound **4** did not display obvious cytotoxicity, which indicated that the oxidative modifications on rings A and B are very important for their mode of actions.

### 2.3. Plausible Biosynthetic Pathways for Compounds ***1**–**8*** Produced by the Mutant Strain

The plausible biogenetic pathway of compounds **1**–**8** was determined based on the bioinformatics analyses (Table S2) and literature surveys. First, the polyketide chain was generated by an iterative Claisen condensation reaction of a starting unit malonyl-CoA and nine extension unit malonyl-CoA under the action of the minimal PKS SpiA, SpiB and SpiC, and was subsequently folded by cyclase SpiE and SpiD to form UWM6 [33,34], a characteristic intermediate of angucyclines; however, if it is spontaneously folded, the by-products **7** and **8** will be produced. Thereafter, UWM6 can achieve the oxidation of C-12 catalyzed by monooxygenase SpiH2 [35] and subsequently undergo reduction at C-6, dehydration and enoyl reduction at C-5 and C-6, and oxidation at C-12b to produce **5**, which could be converted into compounds **1** and **2** via a 6a, 12a-epoxide intermediate. The methanethiol residue of **2** was probably derived from methionine which was incorporated via methane thiol addition to the epoxide. In alternative routes, UWM6 may undergo multi-step catalysis including oxidation, dehydration and oxo-bridge generation to give **3**, **4** and **6** in the presence of relevant endogenous enzymes of the heterologous host.

## 3. Materials and Methods

### 3.1. General Experimental Procedures

High-quality actinobacteria genomes were obtained using methods previously described in the literature [36,37]. Polymerase chain reaction (PCR) was performed using 2 × Phanta Flash Master Mix (P510, Vazyme, Nanjing, China). PCR analyses were conducted with a 2 × Hieff^®^ PCR Master Mix (with dye, Yeasen, Shanghai, China). Shanghai Sangon DNA Technologies (Shanghai, China) offered custom oligonucleotide synthesis and fragment sequencing services.

NMR spectra were measured on an Agilent 500 MHz DD2 spectrometer with tetramethylsilane (TMS) as an internal standard. Optical rotations in MeOH were recorded on a JASCO-1020 digital polarimeter. A Thermo Scientific LTQ Orbitrap XL mass spectrometer (Thermo Fisher Scientific, Waltham, MA, USA) was used to collect HRESIMS data. UV-vis spectra were acquired on the UFLC system (Shimadzu, Tokyo, Japan). The JASCO J-715 spectropolarimeter was used to record the ECD spectra. A UFLC system (Shimadzu, Tokyo, Japan) with a C18 column (Shimadzu, 4.6 mm × 150 mm, 5 μm, 1 mL/min) coupled to an LCMS-2020 mass spectrometer (Shimadzu, Tokyo, Japan) was used to perform LC-MS analyses. Column chromatography (CC) was performed with SiliaSphere C18 (Octadecylsilyl, ODS) monomeric (SiliCycle Inc., Quebec, QC, Canada, 50 μm, 120 A), and Sephadex LH-20 (GE Healthcare, Uppsala, Sweden). MPLC was performed using a C18 column (Welch Materials Inc., Ultimate^®^ XB-C18, 21.2 mm × 250 mm, 5 μm, 10 mL/min).

Prediction and analysis of gene clusters using the online tool antiSMASH. DNA and protein sequence homology analysis using the online tool NCBI.

### 3.2. Materials and Culture Conditions

*Streptomyces* sp. HDN155000 was obtained from a marine sediment sample collected from the South China Sea at coordinates 125°28.550′ E and 29°1.618′ N. The strain was identified via genome sequencing and submitted to GenBank (accession number MN822699). *S. albus* J1074 was grown for 6 days at 28 °C on MS plates with 2% mannitol, 2% soya bean flour, and 1.5% agar. For genome extraction, the strain was inoculated into 250mL Erlenmeyer flasks containing 50 mL of TSB liquid medium (1.7% tryptone, 0.3% peptone, 0.25% glucose, 0.25% KH_2_PO_4_, and 0.3% NaCl) for 4 days.

*Escherichia coli* strains DH10B as the general host for cloning and *E. coli* ET12567/pUZ8002 as the donor in intergeneric conjugation were cultured on Luria–Bertani plates or liquid medium (1% tryptone, 0.5% yeast extract, and 1% NaCl) at 37 °C.

### 3.3. S. albus J1074 Intergeneric Conjugation

The intergeneric conjugation between *E. coli* and *S. albus* J1074 was performed as previously described with some modifications [38]. The culture of the donor *E. coli* ET12567/pUZ8002 containing the heterologous vector p15A::spi was incubated with appropriate antibiotics LB liquid culture medium (containing 50 mg/L kanamycin, 15 mg/L chloramphenicol, and 50 mg/L apramycin,) until the optical density at 600 nm (OD600) of 0.6–0.8. Bacteriophages were collected at 9000 rpm, rinsed three times with 1 mL 2× YT medium [39] to remove antibiotics, and then resuspended in 0.2 mL of 2× YT (containing 10 mM MgCl_2_) as the donor cells. *S. albus* J1074 spores were washed three times and suspended in 2× YT broth at a concentration of 10^9^ per mL. Subsequently, spores obtained in the previous step were heated at 50 °C for 10 min to serve as recipients. Donor and recipient cells were mixed evenly on MS plates (containing 30 mM MgCl_2_ and 30 mM CaCl_2_), and cultivated for 18 h at 30 °C. After incubation, plates were covered with 1 mL of water with apramycin (50 μg/mL) and nalidixic acid (50 μg/mL) and then kept at 30 °C for 4–6 days until exconjugants appeared. The conjugation frequency reached 7.8 × 10^−5^.

### 3.4. Fermentation and LC/LC-MS Analysis

Heterologous expression strains and negative control strains were cultured for 7 days at 28 °C in 500 mL Erlenmeyer flasks with 100 mL of M1 medium (2 g/L peptone, 4 g/L yeast extract, 10 g/L starch), after which the culture products were collected and extracted three times with ethyl acetate. The organic phase was evaporated, and the residue was re-dissolved in 150 μL MeOH, which was analyzed using HPLC (C18 column, Shimadzu, 4.6 mm × 150 mm, 5 μm, 1 mL/min), revealing a considerable change in metabolite synthesis in the heterologous expression strain J1074::spi (Figure 1).

For the isolation of secondary metabolites, we scaled up cultures with the 500 mL Erlenmeyer flask (total 20 L) according to the conditions used for small-scale analyses. The fermentation supernatant was extracted with ethyl acetate three times and then evaporated to dryness, obtaining a total of 11.3 g of crude product.

### 3.5. Extraction, Isolation, and Purification

The crude extract was eluted using a step gradient of MeOH-H_2_O using metabolite detection on a C18 column, yielding seven subfractions (Fr.1-Fr.7, 30% to 100%). Fr.2 was purified by semi-preparative HPLC (33:67 MeOH-H_2_O, 3 mL/min) to generate compounds **1** (7.1 mg, t_R_ = 15.1 min) and **6** (3.1 mg, t_R_ = 16.7 min). Fr.3 was purified by a semi-preparative C18 HPLC column (47:53 MeOH-H_2_O), producing compound **3** (6.4 mg, t_R_ = 14.7 min). Fr.4 was separated using preparative C18 HPLC column (58:42 MeOH-H_2_O) to yield two sub-fractions (Fr.4.1 and Fr.4.2). Then, Fr.4.1 and Fr.4.2 were purified by semi-preparative HPLC (53:47, 60:40, MeOH-H_2_O, 3 mL/min) to provide compounds **4** (4.7 mg, t_R_ = 18.1 min), **5** (3.4 mg, t_R_ = 20.7 min), **7** (3.8 mg, t_R_ = 16.9 min), and **8** (3.0 mg, t_R_ = 19.4 min), respectively. Fr.5 was applied to a Sephadex LH-20 column and eluted with methanol, obtaining compound **2** (5.6 mg).

Angumycinone Z (**1**): pale brown powder, ${\left[\alpha \right]}_{D}^{25}$ + 19.5 (*c* 0.03, CH_3_OH); UV (DAD) λ_max_ 208 nm, 250 nm, 327 nm, 386 nm; CD (MeOH) λ_max_ (∆ε) 210 (−19.06), 243 (+33.91), 283 (−4.88), 302 (+3.13), 336 (−3.39), 376 (+5.21); ^1^H and ^13^C NMR data, see Table 1 and Table 2; negative ion HRESIMS *m*/*z* 373.0916 [M − H]^−^ (calcd. for C_19_H_17_O_7_^−^, 373.0928).

WS009 Z (**2**): even brown amorphous powder, ${\left[\alpha \right]}_{D}^{25}$ + 55.8 (*c* 0.03, CH_3_OH); UV (DAD) λ_max_ 249, 302, 352; ^1^H and ^13^C NMR data, see Table 1 and Table 2; positive ion HRESIMS *m*/*z* 405.0996 [M + H]^+^ (calcd. for C_20_H_21_O_7_S^+^, 405.1003).

Angumycinone X (**3**): yellowish, solid powder, ${\left[\alpha \right]}_{D}^{25}$ + 79.1 (*c* 0.03, CH_3_OH); UV (DAD) λ_max_ 206, 238, 309, 364; CD (MeOH) λ_max_ (∆ε) 211 (−12.77), 242 (+25.31), 281 (−3.28), 300 (+2.63), 334 (−2.96), 372 (+4.21); ^1^H and ^13^C NMR data, see Table 1 and Table 2; negative ion HRESIMS *m*/*z* 355.0821 [M − H]^−^ (calcd. for C_19_H_15_O_7_^−^, 355.0823).

Angumycinone T (**4**): brown amorphous powder, ${\left[\alpha \right]}_{D}^{25}$ + 31.0 (*c* 0.03, CH_3_OH); UV (DAD) λ_max_ 206, 236, 298, 350; CD (MeOH) λ_max_ (∆ε) 207 (+3.30), 227 (+19.67), 243 (−11.30), 274 (+14.31), 332 (−2.67), 370 (+2.94); ^1^H and ^13^C NMR data, see Table 1 and Table 2; negative ion HRESIMS *m*/*z* 355.0815[M − H]^−^ (calcd. for C_19_H_15_O_7_^−^, 355.0823)

### 3.6. NMR and ECD Calculations

Conformation searches based on molecular mechanics with MMFF force fields were performed for stereoisomers to obtain stable conformers. The Gaussian 16 programme package [40] used the density functional theory (DFT) approach at the B3LYP/6-31G(d) level to optimize all conformers. Gauge Independent Atomic Orbital (GIAO) calculations of their ^1^H and ^13^C NMR chemical shifts were performed utilizing density functional theory (DFT) at the mPW1PW91/6-311+G(d,p) level with the PCM model in DMSO [41]. The calculated NMR data of these conformers were averaged using the Boltzmann distribution theory and their respective Gibbs free energy. The ^1^H and ^13^C NMR chemical shifts for TMS were computed using the same methodology and served as a reference. Following calculation, the experimental and calculated data were compared using linear correlation coefficients (R^2^) and the improved probability DP4+ technique [42].

All stable conformers were further optimized at the B3LYP/6-31G(d)-GD3BJ level with the Gaussian 16 programme package. The ECD was computed using time-dependent density functional theory (TDDFT) at a B3LYP/6-311+G(d,p) level with the IEFPCM model. The calculated ECD curves were all generated using the SpecDis 1.71 computer package and the calculated ECD data of all conformers were Boltzmann averaged using Gibbs free energy [43].

### 3.7. Cytotoxicity Assay

The cytotoxic assay involved five human cancer cell lines: K562 (using the MTT method), ASPC-1, NCI-H446/EP, MDA-MB-231, and L-02 (using the SRB method). Adriamycin (ADM) was employed as a positive control. The detailed procedures for biological testing were conducted as previously stated [20,44]. The cancer cell lines are purchased from the National Collection of Authenticated Cell Cultures of China (Shanghai).

## 4. Conclusions

Secondary metabolites from actinobacteria have long been a valuable source of natural medicines [45]. However, the success rate for identifying actinobacteria-derived novel compounds is limited by gene cluster silencing or low expression under laboratory conditions, especially for micro-organisms from special habitats such as marine actinobacteria. In this study, the type II PKS gene cluster *spi* derived from *Streptomyces* sp. HDN155000 was successfully heterologously expressed in *S. albus* J1074, and a total of eight compounds were obtained through further fermentation, isolation, and purification. Based on the literature and our previous study, we proposed a plausible biosynthetic pathway leading to those new compounds [4,34]. Generally, compounds **3**–**6** were directly produced from UWM6 by a multi-step enzymatic reaction involving oxidation, dehydration, enoyl reduction, etc. Compounds **1** and **2** were derived from compound **5** via dehydrogenation, hydrolysis, methane thiol addition, and other processes. Angucyclines are a chemical family that has been extensively explored in actinobacteria since the first structure was described in 1965 in *Streptomyces rimosus* [2]. More than 400 cases have been recorded, with a range of skeleton modifications; however, compound **4** is the first example of oxidized angucyclines with an oxo-bridge between C-12a and C-4 [3]. Compound **3** showed cytotoxic to the K562 and NCI-H446/EP cell lines, providing an alternative lead compound for the study of human chronic myeloid leukemia and lung cancer. The above data enrich our understanding of the type II PKS pathways and related aromatic products, and also indicate heterologous expression as a promising strategy for maximizing the metabolic capability of *Streptomyces*.

## Figures and Tables

**Table 1:** ^1^H Spectroscopic Data for **1**–**4** in DMSO-*d*_6_ (500 MH_Z_).

No.	1	2	3	4
	*δ*_H_ (*J* in Hz)	*δ*_H_ (*J* in Hz)	*δ*_H_ (*J* in Hz)	*δ*_H_ (*J* in Hz)
2	5.71, s	5.70, s	5.97, s	5.83, s
4	2.89, d (18.0)	2.94, d (18.0)	3.06, d (18.0)	3.98, d (1.3)
	2.06, d (18.0)	2.13, d (18.0)	2.02, d (18.0)	-
5	1.98, m	2.07, m	1.76, m	1.98, m
	1.60, m	1.63, m	1.35, m	-
6	2.28, m	2.36, m	2.53, m	2.24, m
	1.74, m	1.84, m	2.38, m	2.14, m
9	7.34, d (8.0)	7.35, d (8.0)	7.31, m	7.35, dd (8.0, 1.0)
10	7.76, t (8.0)	7.73, t (8.0)	7.69, dd (8.2, 7.6)	7.76, dd (8.0, 7.6)
11	7.60, d (8.0)	7.58, d (8.0)	7.34, m	7.34, dd (7.6, 1.0)
12b	-	-	-	3.14, s
4a-OH	6.32, s	-	2.54, s	5.59, s
6a-OH	6.03, s	-	-	7.05, s
12a-OH	7.01, s	-	-	-
12b-OH	5.66, s	-	-	-
8-OH	11.10, s	11.17, s	11.06, s	11.72, s
13	1.93, s	1.96, s	1.81, s	2.33, d (1.2)
S-CH_3_	-	1.67, s	-	-

**Table 2:** ^13^C Spectroscopic Data for **1**–**4** in DMSO-*d*_6_ (125 MH_Z_).

No.	1	2	3	4
	*δ*_C_, Type	*δ*_C_, Type	*δ*_C_, Type	*δ*_C_, Type
1	192.8, C	193.5, C	196.7, C	195.6, C
2	122.9, CH	122.7, CH	122.6, CH	124.6, CH
3	158.2, C	159.4, C	156.1, C	162.4, C
4	41.8, CH_2_	41.6, CH_2_	38.2, CH_2_	85.1, CH
4a	75.5, C	75.7, C	65.1, C	81.6, C
5	28.2, CH_2_	28.8, CH_2_	25.8, CH_2_	32.6, CH_2_
6	21.6, CH_2_	17.9, CH_2_	20.1, CH_2_	26.8, CH_2_
6a	77.6, C	61.2, C	74.2, C	78.0, C
7	200.5, C	195.5, C	194.5, C	201.6, C
7a	116.3, C	115.4, C	114.7, C	114.9, C
8	159.9, C	160.0, C	159.5, C	161.1, C
9	123.6, CH	124.4, CH	123.9, CH	123.7, CH
10	136.2, CH	136.0, CH	136.4, CH	137.7, CH
11	118.9, CH	119.0, CH	118.9, CH	118.3, CH
11a	133.0, C	131.5, C	132.7, C	135.3, C
12	194.1, C	191.0, C	189.6, C	192.1, C
12a	76.4, C	78.4, C	67.5, C	92.5, C
12b	76.2, C	75.7, C	73.7, C	52.7, CH
13	23.6, CH_3_	23.7, CH_3_	23.6, CH_3_	26.3, CH_3_
S-CH_3_	-	13.3, CH_3_	-	-

## Data Availability

The data given in this research are available in this article and the Supplementary Materials.

## Supplementary Materials

The following supporting information can be downloaded at: https://www.mdpi.com/article/10.3390/md22110480/s1, Figure S1: Restriction to validate heterologous expression plasmid p15A-spi and PCR analysis for confirming the heterologous strain J1074::spi; Figure S2: Comparative analysis of metabolites from two heterologous hosts, *S. albus* J1074 and *S. coelicolor* A3; Figure S3: ^13^C NMR calculation results of four possible isomers of **3**; Figures S4–S10: NMR and HRESIMS spectra of compound **1**; Figures S11–S16: NMR and HRESIMS spectra of compound **2**; Figures S17–S22: NMR and HRESIMS spectra of compound **3**; Figures S23–S29: NMR and HRESIMS spectra of compound **4**; Table S1: The primers used in this study; Table S2: Deduced function of 25 genes in *spi*; Table S3–S6: Calculated ^13^C NMR results for **3a**, **3b**, **3c**, **3d**; Table S7: DP4+ analysis results of **3a** (Isomer 1), **3b** (Isomer 2), **3c** (Isomer 3) and **3d** (Isomer 4).
