# Supplementary material for: Heterologous Expression of Type II PKS Gene Cluster Leads to Diversified Angucyclines in Streptomyces albus J1074
Source: Mar Drugs. 2024 Oct 22;22(11):480. doi: 10.3390/md22110480 (PMC11595736; doi:10.3390/md22110480)

Heterologous Expression of Type II PKS Gene Cluster Leads to Diversified Angucyclinones in *Streptomyces albus* J1074

Contents

Section S1. Construction of heterologous expression strain J1074::spi. **2**

Section S2. Calculational details for 3 .... **4**

S2.1. Computational details for compound 3 (NMR) **4**

S2.2. Computational details for compound 3 (ECD) **11**

Section S3. NMR and HRESIMS spectra of 1 .... **12**

Section S4. NMR and HRESIMS spectra of 2 .... **16**

Section S5. NMR and HRESIMS spectra of 3 .... **19**

Section S6. NMR and HRESIMS spectra of 4 .... **22**

Section S1. Construction of heterologous expression strain J1074::spi.

Table S1. The primers used in this study (5’ to 3’).

Primers	Sequences
spi-F	CGCGCCGCGTGCGGCCTTGTGCGGGACGTCCTCCCACCGCAGTTCCTGGC CCGTCTCCTCGGCGATGATCTGCAATTGctcgagagatccgaaaaccc
spi-R	CCTTCTCAGATCTCCGTGATGTTTCTTCAGAGATCGACACGTTCGGAACGG GGAGGTCAAGCAAGCGTCAAGGAAACTtcttattcggccttgaattg
YZ-spi-F	GTGCCGCCCTGGCTGTATGAG
YZ-spi-R	GGCGCTTGCTGAGCCATGATC

Note: The homologous arms are represented by capital letters.

Table S2. Deduced function of 25 genes in *spi*.

Gene	Size[a]	Putative Function	Closest Homolog (Origin, Accession No.)	Identity (%)
spi H1	238	antibiotic biosynthesis monooxygenase	Streptomyces sp. HNM0574, WP_169443009.1	112/202(55%)
spi L	438	DUF1205 domain-containing protein(glycosyltransferase)	Streptomyces abyssalis, WP_070011378.1	310/426(73%)
spi M	257	Methyltransferase	Streptomyces griseus, QDG00806.1	250/250(100%)
spi N	377	glycosyl transferase	Streptomyces griseus, QDG00807.1	376/377(99%)
spi O	539	drug resistance transporter	Streptomyces griseus, QDG00808.1	539/539(100%)
spi P	277	N, N-dimethyltransferase	Streptomyces griseus, QDG00809.1	276/277(99%)
spi Q	385	NDP-hexose aminotransferase	Streptomyces griseus, QDG00810.1	309/309(100%)
spi R	196	NDP-hexose 35-epimerase	Streptomyces griseus, QDG00811.1	196/196(100%)
spi S	355	glucose-1-phosphate thymidylyltransferase	Streptomyces griseus, QDG00812.1	292/293(99%)
spi T	338	dTDP-glucose 46-dehydratase	Streptomyces griseus, QDG00813.1	321/322(99%)
spi U	434	NDP-hexose 34-dehydratase	Streptomyces griseus, QDG00814.1	434/434(100%)
spi V	460	NDP-hexose 23-dehydratase	Streptomyces griseus, QDG00815.1	458/460(99%)
spi W	319	dNDP-hexose 3-ketoreductase	Streptomyces griseus， QDG00816.1	297/297(100%)
spi I	407	methyltransferase	Streptomyces griseus， QDG00817.1	406/407(99%)
spi J	327	Adenosine kinase	Streptomyces griseus， QDG00818.1	288/289(99%)
spi K	289	methylenetetrahydrofolate reductase	Streptomyces griseus， QDG00819.1	289/289(100%)
spi G	298	thioesterase	Streptomyces griseus， QDG00820.1	298/298(100%)
spi H2	775	oxygenase-reductase	Streptomyces griseus， QDG00821.1	775/778(99%)
spi E	311	polyketide synthase	Streptomyces griseus， QDG00822.1	311/311(100%)
spi F	260	polyketide ketoreductase	Streptomyces griseus， QDG00823.1	260/260(100%)
spi A	89	PP-binding	Streptomyces griseus， QDG00824.1	89/89(100%)
spi B	405	ketosynthase chain length factor	Streptomyces griseus, QDG00825.1	403/405(99%)
spi C	353	polyketide synthase	Streptomyces griseus, QDG00826.1	353/353(100%)
spi D	109	TcmI family type II polyketide cyclase	Streptomyces griseus, QDG00827.1	109/109(100%)
spi H2	510	FAD-binding protein	Streptomyces griseus, QDG00828.1	510/510(100%)

Figure S1. Restriction to validate heterologous expression plasmid p15A-spi and PCR analysis for confirming the heterologous strain J1074::spi.

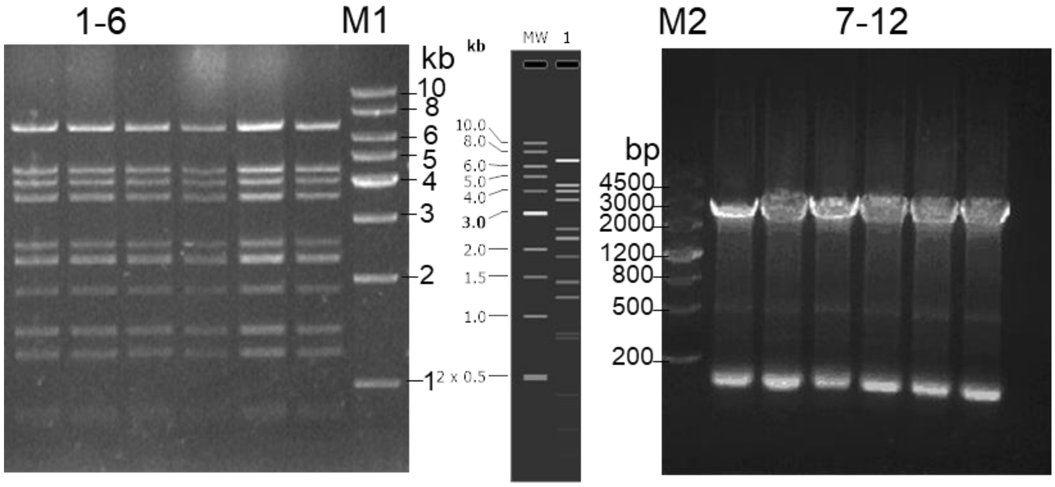

1-6: p15A-spi
7-12: J1074::spi
M1: 1kb DNA ladder
M2: marker III

Figure S2. Comparative analysis of metabolites from two heterologous hosts, *S. albus* J1074 and *S. coelicolor* A3.

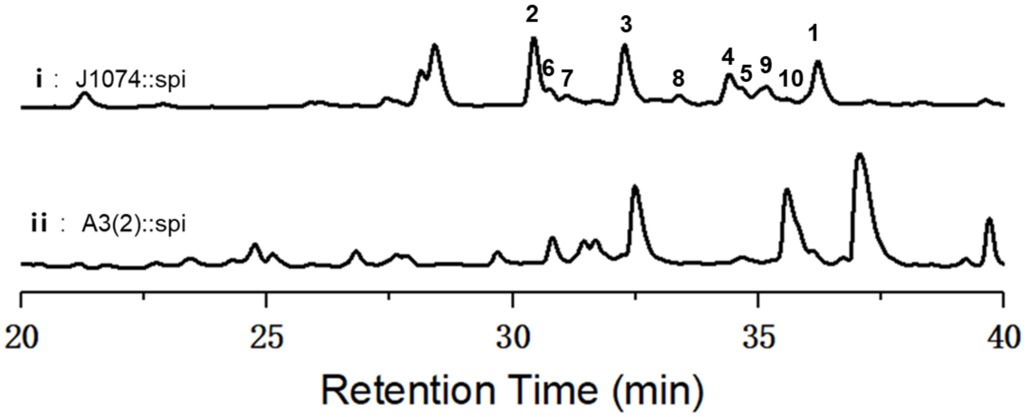

Section S2. Calculational details for 3

S2.1. Computational details for compound 3 (NMR)

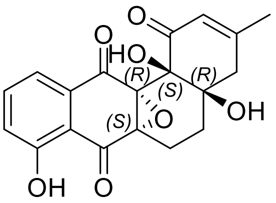

3a

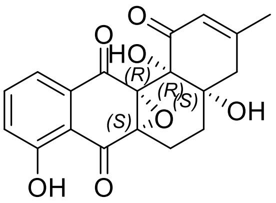

3b

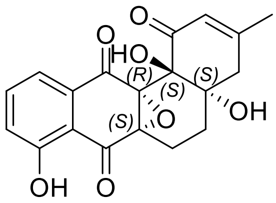

3c

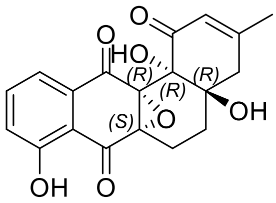

3d

Conformation search based on molecular mechanics with MMFF force fields (Spartan′14, Wavefunction, Inc.) were performed for **3a**, **3b**, **3c**, and **3d** stable conformers within 20 kJ/mol, respectively. All these conformers were further optimized by the density functional theory method at the B3LYP/6-31G(d) level by Gaussian 16 program package. Gauge Independent Atomic Orbital (GIAO) calculations of their ^{1}H and ^{13}C NMR chemical shifts of stable conformers with populations higher than 1% using density functional theory (DFT) at the mPW1PW91/6-311+G(d,p) level with the PCM model in DMSO. The calculated NMR data of these conformers were averaged according to the Boltzmann distribution theory and their relative Gibbs free energy. The ^{1}H and ^{13}C NMR chemical shifts for TMS were also calculated by the same procedures and used as the reference. After calculation, the experimental and calculated data were evaluated by linear correlation coefficients (R^2) and the improved probability DP4+ method.

Table S3. Calculated ^{13}C NMR results for **3a**

No.	**3aA**	$\delta_{Calcd}{}^{a}$	δ_{Exp}	$\delta_{Corr}{}^{b}$	Relative errors^{c}
1	42.7825	146.31	136.4	135.7	0.7
2	55.2507	133.84	123.9	124.1	-0.2
3	18.5188	170.57	159.5	158.3	1.2
4	68.9625	120.13	114.7	111.3	3.4
5	49.3145	139.78	132.7	129.6	3.1
6	61.1395	127.95	118.9	118.6	0.3
7	-19.4587	208.55	194.5	193.7	0.8
8	115.774	73.32	74.2	67.7	6.5
9	123.1292	65.96	67.5	60.8	6.7
10	-20.3554	209.45	189.6	194.5	-4.9
11	166.9645	22.13	20.1	19.9	0.2
12	160.586	28.51	25.8	25.9	-0.1
13	112.3826	76.71	65.1	70.8	-5.7
14	107.1074	81.99	73.7	75.7	-2.0
15	141.993	47.10	38.2	43.2	-5.0
16	9.6043	179.49	156.1	166.6	-10.5
17	58.2889	130.80	122.6	121.2	1.4
18	-15.2393	204.33	196.7	189.8	6.9
19	160.1472	28.95	23.6	26.3	-2.7
Population	100%			RMSD	4.39

^{a}Weighted average from the calculated shifts; ^{b}Obtained by linear fit δexp versus δcalcd; $^{c}\Delta\delta = \delta_{exp} - \delta_{corr}$.

Table S4. Calculated ^{13}C NMR results for **3b**

No.	**3bA**	$\delta_{Calcd}{}^{a}$	δ_{Exp}	$\delta_{Corr}{}^{b}$	Relative errors^{c}
1	42.5711	146.52	136.4	135.7	0.7
2	54.5279	134.56	123.9	124.4	-0.5
3	17.3035	171.79	159.5	159.5	0.0
4	69.3291	119.76	114.7	110.4	4.3
5	51.4045	137.69	132.7	127.3	5.4
6	61.4531	127.64	118.9	117.9	1.0
7	-18.8672	207.96	194.5	193.6	0.9
8	118.3216	70.77	74.2	64.3	9.9
9	118.3738	70.72	67.5	64.2	3.3
10	-15.0848	204.18	189.6	190.0	-0.4
11	166.6374	22.46	20.1	18.7	1.4
12	157.2144	31.88	25.8	27.6	-1.8
13	108.3237	80.77	65.1	73.7	-8.6
14	104.6475	84.45	73.7	77.2	-3.5
15	140.6253	48.47	38.2	43.2	-5.0
16	8.6335	180.46	156.1	167.6	-11.5
17	59.124	129.97	122.6	120.1	2.5
18	-19.3664	208.46	196.7	194.0	2.7
19	160.6265	28.47	23.6	24.4	-0.8
Population	100%			RMSD	4.72

^{a}Weighted average from the calculated shifts; ^{b}Obtained by linear fit δexp versus δcalcd; $^{c}\Delta\delta = \delta_{exp} - \delta_{corr}$.

Table S5. Calculated ^{13}C NMR results for **3c**

No.	**3cA**	**3cB**	**3cC**	δ_{Calcd}[a]	δ_{Exp}	δ_{Corr}[b]	Relative errors[c]
1	42.3297	42.8426	42.8068	146.76	136.4	136.1	0.3
2	56.1002	56.1846	56.3239	132.99	123.9	123.2	0.7
3	18.4443	18.6424	18.6705	170.65	159.5	158.7	0.8
4	68.9689	68.8599	68.9038	120.12	114.7	111.0	3.7
5	48.1802	48.0886	47.7637	140.91	132.7	130.6	2.1
6	61.9489	62.1046	62.2048	127.14	118.9	117.7	1.2
7	-17.302	-19.1728	-19.3824	206.40	194.5	192.4	2.1
8	115.4989	116.5672	117.0396	73.59	74.2	67.2	7.0
9	117.7076	119.6304	119.3357	71.38	67.5	65.1	2.4
10	-20.2432	-22.2153	-22.2109	209.35	189.6	195.1	-5.5
11	167.6887	167.7635	167.66	21.40	20.1	18.0	2.1
12	156.6373	157.2861	155.5407	32.45	25.8	28.4	-2.6
13	107.5054	107.3773	106.3766	81.59	65.1	74.7	-9.6
14	109.2355	108.1955	108.6002	79.86	73.7	73.1	0.6
15	143.2405	141.533	140.7433	45.86	38.2	41.0	-2.8
16	12.4704	11.1032	14.6061	176.63	156.1	164.3	-8.2
17	58.8449	57.4168	58.121	130.26	122.6	120.6	2.0
18	-16.7958	-15.883	-16.2236	205.88	196.7	191.9	4.8
19	160.5816	160.0858	160.6097	28.51	23.6	24.7	-1.1
Population	99.48%	0.49%	0.03%			RMSD	4.10

[a]Weighted average from the calculated shifts; [b]Obtained by linear fit δexp versus δcalcd; [c]$\Delta\delta = \delta_{exp} - \delta_{corr}$.

Table S6. Calculated ^{13}C NMR results for **3d**

No.	**3dA**	**3dB**	$\delta_{Calcd}{}^{a}$	δ_{Exp}	$\delta_{Corr}{}^{b}$	Relative errors^{c}
1	42.4489	42.4986	146.60	136.4	136.4	0.0
2	55.1996	55.0305	134.04	123.9	124.5	-0.6
3	17.2195	17.2405	171.85	159.5	160.3	-0.8
4	68.785	68.7933	120.30	114.7	111.4	3.3
5	50.2018	50.5012	138.63	132.7	128.8	3.9
6	61.946	61.8839	127.20	118.9	118.0	0.9
7	-19.2679	-19.1084	208.22	194.5	194.8	-0.3
8	116.8895	116.7569	72.32	74.2	65.9	8.3
9	117.8749	118.1002	71.02	67.5	64.7	2.8
10	-11.9886	-12.1402	201.21	189.6	188.2	1.4
11	165.0606	165.5628	23.59	20.1	19.7	0.4
12	158.1686	159.2725	29.96	25.8	25.7	0.1
13	108.009	109.144	80.09	65.1	73.3	-8.2
14	106.6579	105.8345	83.16	73.7	76.2	-2.5
15	142.1161	142.7917	46.38	38.2	41.3	-3.1
16	15.8236	13.1937	175.58	156.1	163.9	-7.8
17	58.087	57.1194	131.85	122.6	122.4	0.2
18	-18.4536	-18.7202	207.78	196.7	194.4	2.3
19	161.2903	160.904	28.14	23.6	24.0	-0.4
Population	12%	88%			RMSD	3.66

[a]Weighted average from the calculated shifts; [b]Obtained by linear fit δexp versus δcalcd; $^{c}\Delta\delta = \delta_{exp} - \delta_{corr}$.

Figure S3. ^{13}C NMR calculation results of four possible isomers of **3**. (A) Linear correlation plots of predicted versus experimental ^{13}C NMR chemical shifts. (B) Relative errors between the predicted ^{13}C NMR chemical shifts of two potential structures and recorded ^{13}C NMR data.

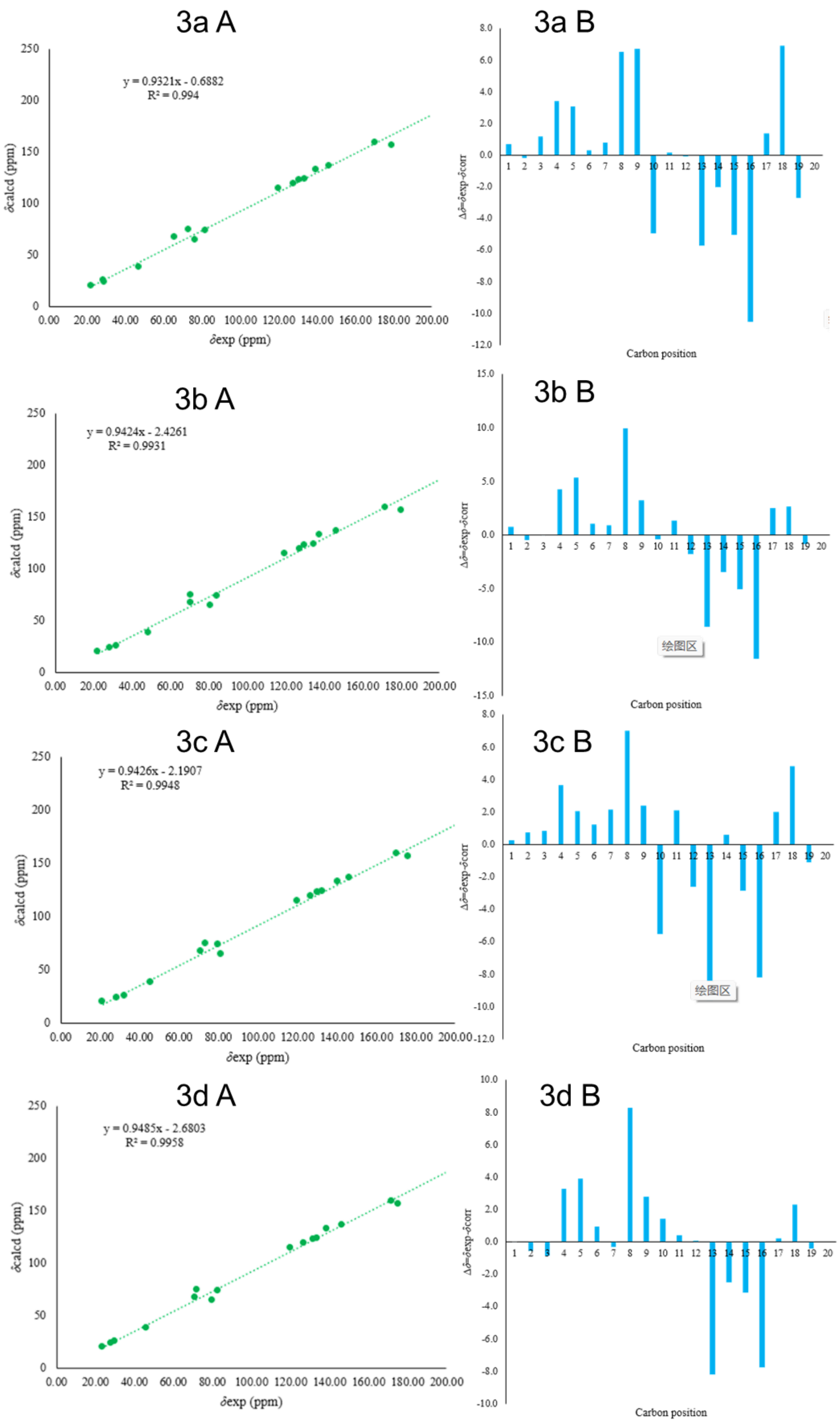

Table S7. DP4+ analysis results of **3a** (Isomer 1), **3b** (Isomer 2), **3c** (Isomer 3) and **3d** (Isomer 4)

A	B	C	D	E	F	G	H
Functional		Solvent?		Basis Set		Type of Data	
mPW1PW91		PCM		6-31+G(d,p)		Shielding Tensors	
		DP4+	0.09%	0.00%	0.01%	99.90%	-
Nuclei	sp2?	xperimenta	Isomer 1	Isomer 2	Isomer 3	Isomer 4	Isomer 5
C	x	136.4	42.8	42.6	42.3	42.5	
C	x	123.9	55.3	54.5	56.1	55.1	
C	x	159.5	18.5	17.3	18.4	17.2	
C	x	114.7	69.0	69.3	69.0	68.8	
C	x	132.7	49.3	51.4	48.2	50.5	
C	x	118.9	61.1	61.5	61.9	61.9	
C	x	194.5	-19.5	-18.9	-17.3	-19.1	
C		74.2	115.8	118.3	115.5	116.8	
C		67.5	123.1	118.4	117.7	118.1	
C	x	189.6	-20.4	-15.1	-20.3	-12.1	
C		20.1	167.0	166.6	167.7	165.5	
C		25.8	160.59	157.21	156.64	159.14	
C		65.1	112.38	108.32	107.50	109.00	
C		73.7	107.11	104.65	109.23	105.94	
C		38.2	141.99	140.63	143.23	142.71	
C	x	156.1	9.60	8.63	12.46	13.52	
C	x	122.6	58.29	59.12	58.84	57.24	
C	x	196.7	-15.24	-19.37	-16.79	-18.69	
C		23.6	160.15	160.63	160.58	160.95	

A	B	C	D	E	F	G	H
Functional		Solvent?		Basis Set		Type of Data	
mPW1PW91		PCM		6-31+G(d,p)		Shielding Tensors	
		Isomer 1	Isomer 2	Isomer 3	Isomer 4	Isomer 5	Isomer 6
sDP4+ (H data)		-	-	-	-	-	-
sDP4+ (C data)		0.00%	0.00%	0.02%	99.98%	-	-
sDP4+ (all data)		0.00%	0.00%	0.02%	99.98%	-	-
uDP4+ (H data)		-	-	-	-	-	-
uDP4+ (C data)		97.85%	0.32%	0.62%	1.21%	-	-
uDP4+ (all data)		97.85%	0.32%	0.62%	1.21%	-	-
DP4+ (H data)		-	-	-	-	-	-
DP4+ (C data)		0.09%	0.00%	0.01%	99.90%	-	-
DP4+ (all data)		0.09%	0.00%	0.01%	99.90%	-	-

S2.2. Computational details for compound 3 (ECD)

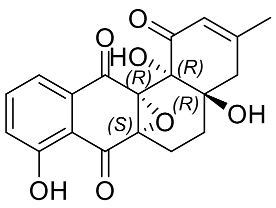

3d

Conformation search based on molecular mechanics with MMFF force fields (Spartan'14, Wavefunction, Inc.) were performed for **3d** stable conformers within 20 kJ/mol, respectively. All these conformers were further optimized by the density functional theory method at the B3LYP/6-31G(d) level by Gaussian 16 program package. The ECD of stable conformers with populations higher than 1% were calculated using density functional theory (TDDFT) at B3LYP/6-31+G(d) level in methanol with IEFPCM model. The calculated ECD curves were all generated using SpecDis 1.71 with $\sigma = 0.30$ eV, and UV shift -18 nm, respectively.

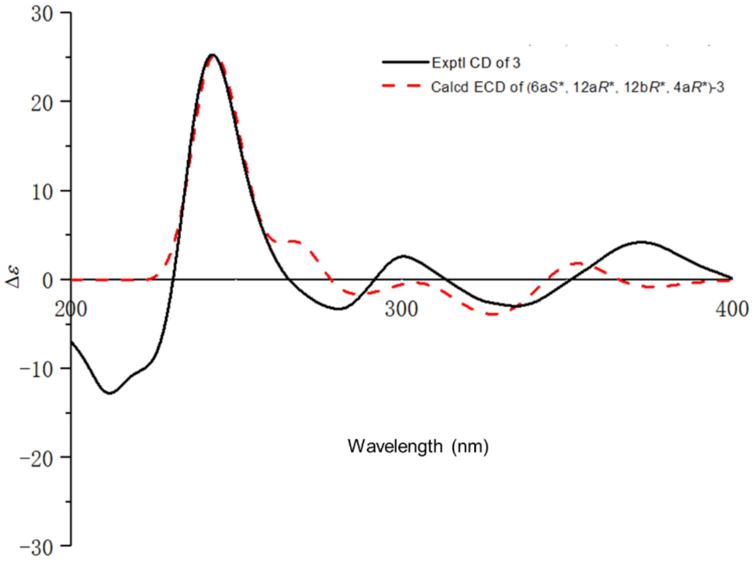

Section S3. NMR and HRESIMS spectra of 1

Figure S4. ^{1}H NMR of **1** in DMSO-d_6

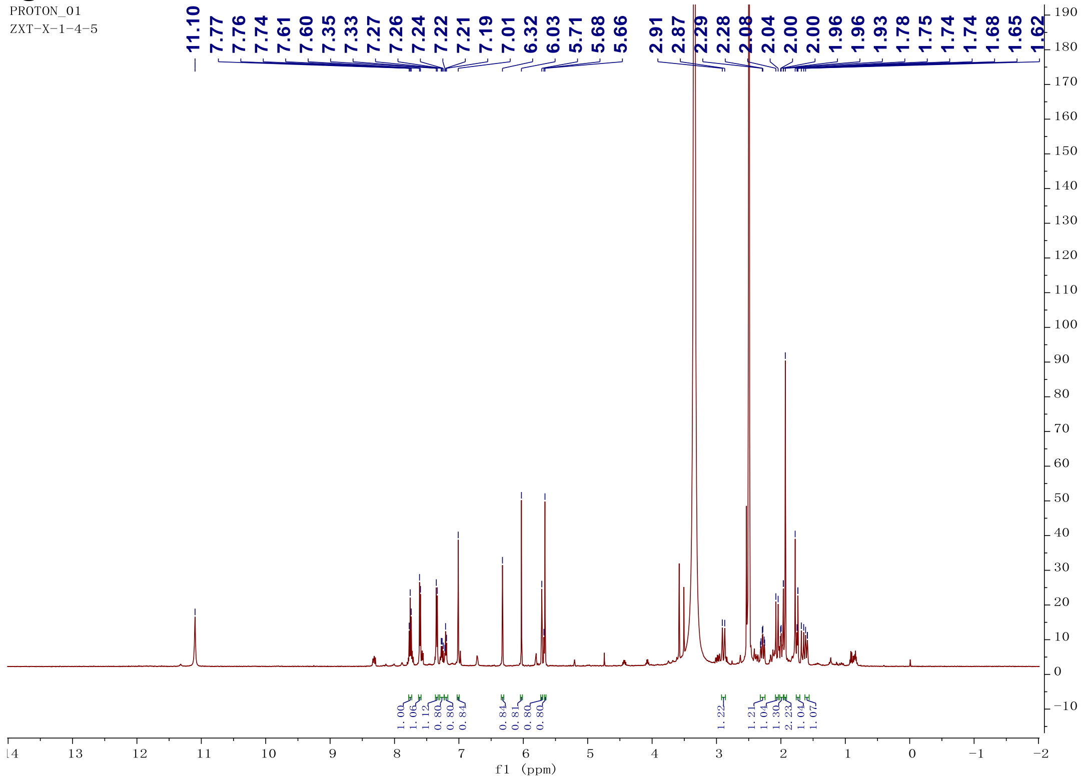

Figure S5. 13C NMR of **1** in DMSO-d_6

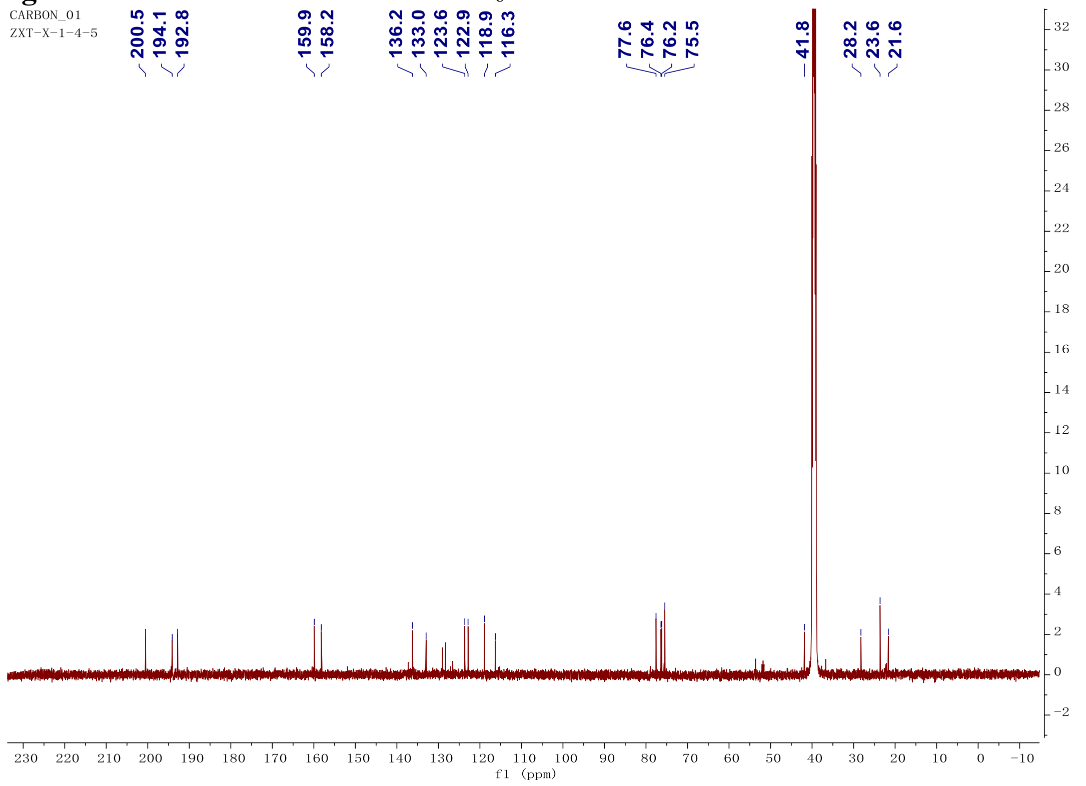

Figure S6. HSQC of **1** in DMSO-d_6

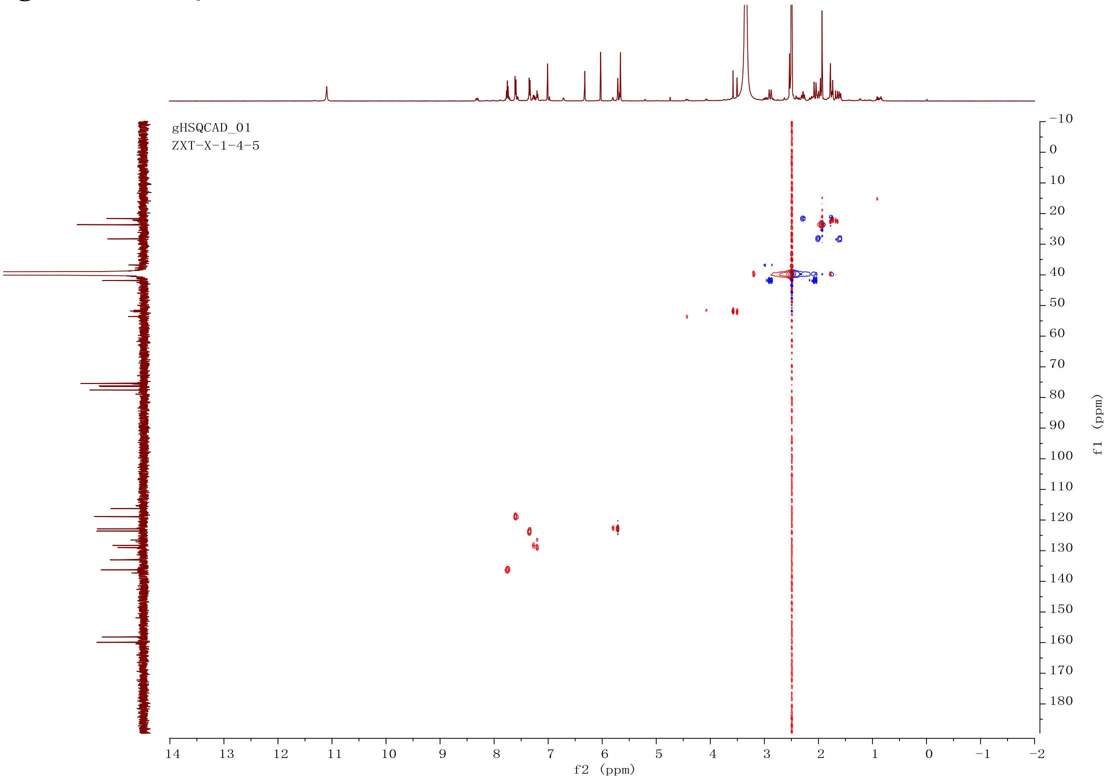

Figure S7. HMBC of **1** in DMSO- d_6

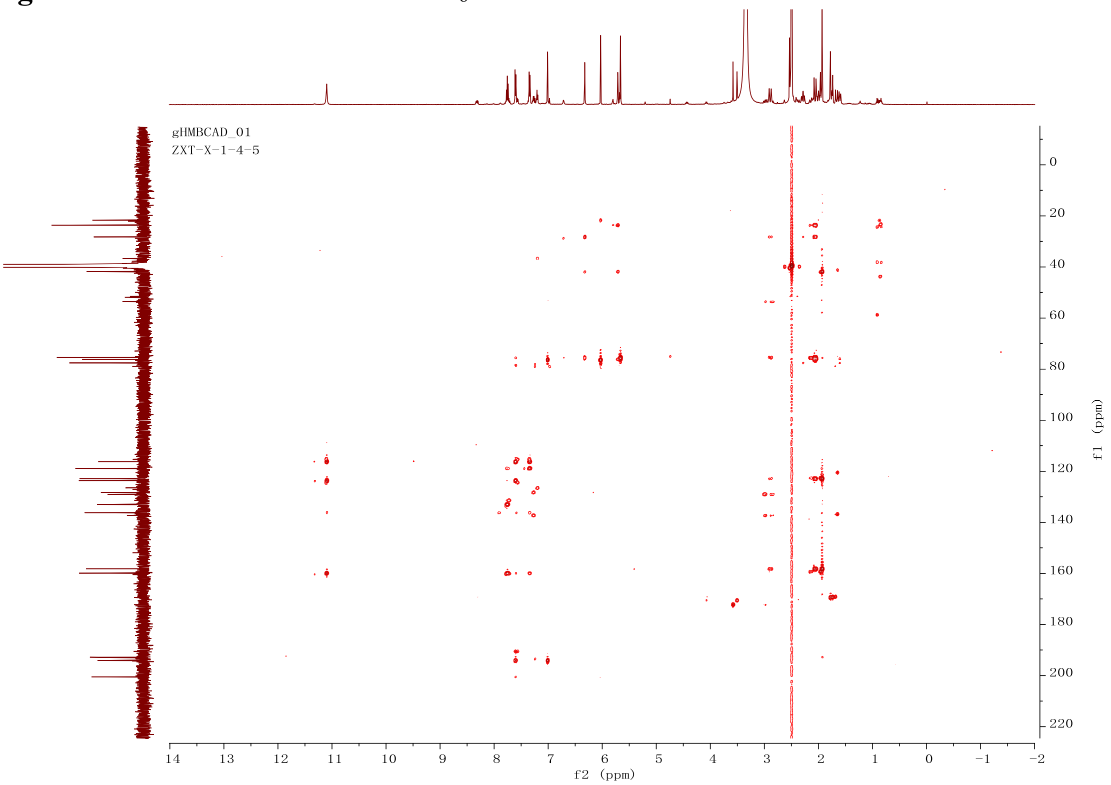

Figure S8. ^{1}H-^{1}H COSY of **1** in DMSO-d_6

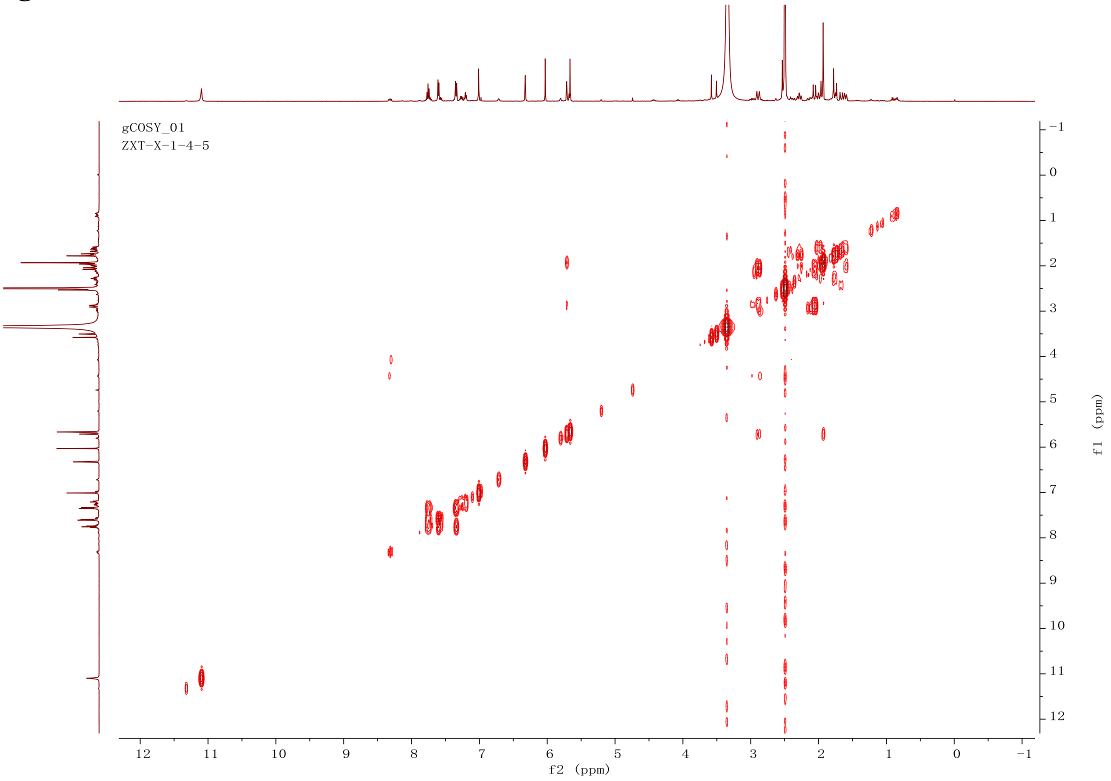

Figure S9. NOESY of **1** in DMSO- d_6

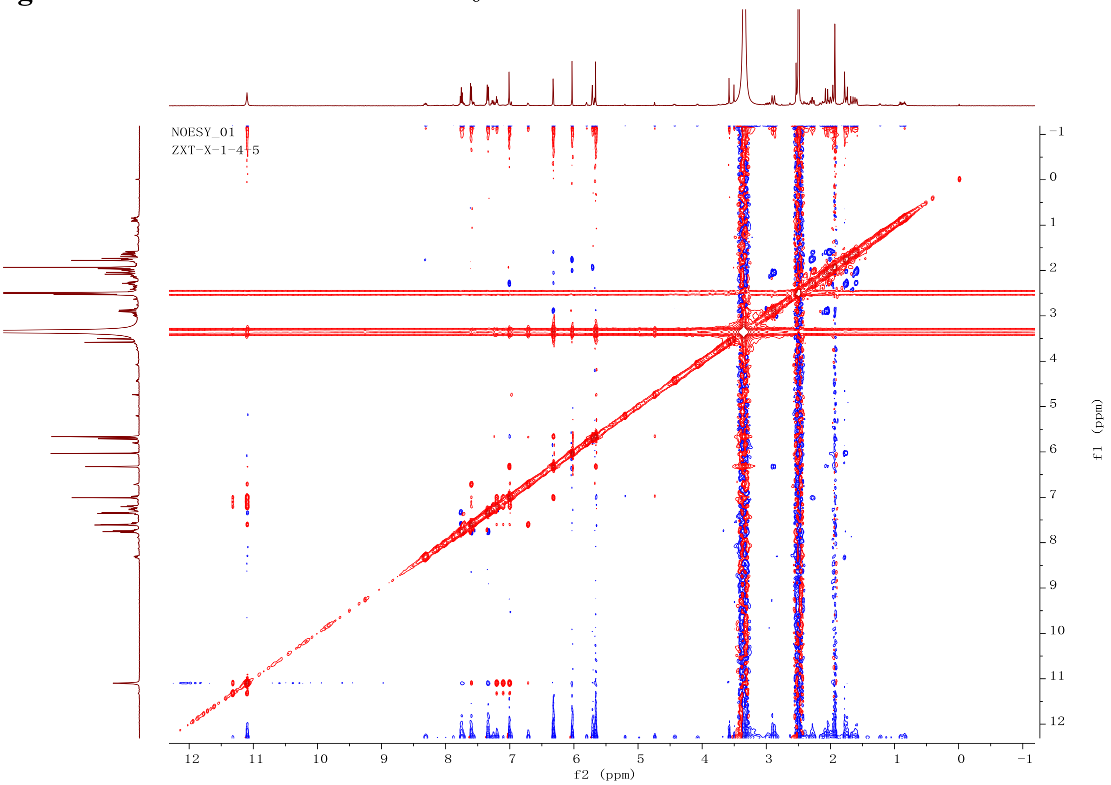

Figure S10. HRESIMS of **1**

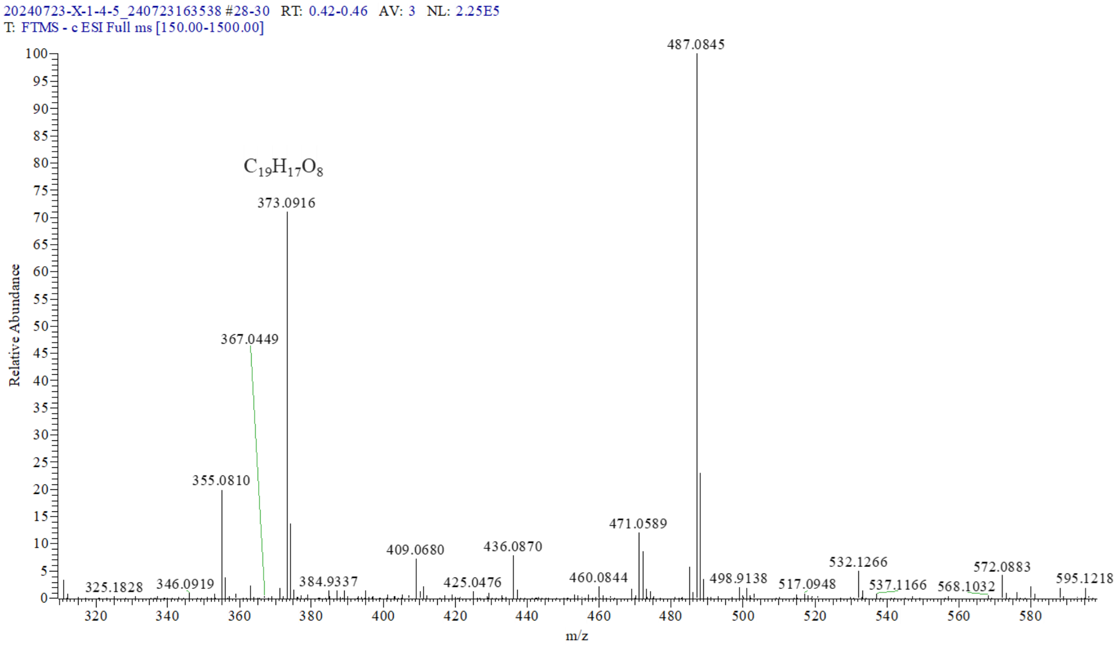

Section S4. NMR and HRESIMS spectra of 2

Figure S11. ^{1}H NMR of **2** in DMSO-d_6

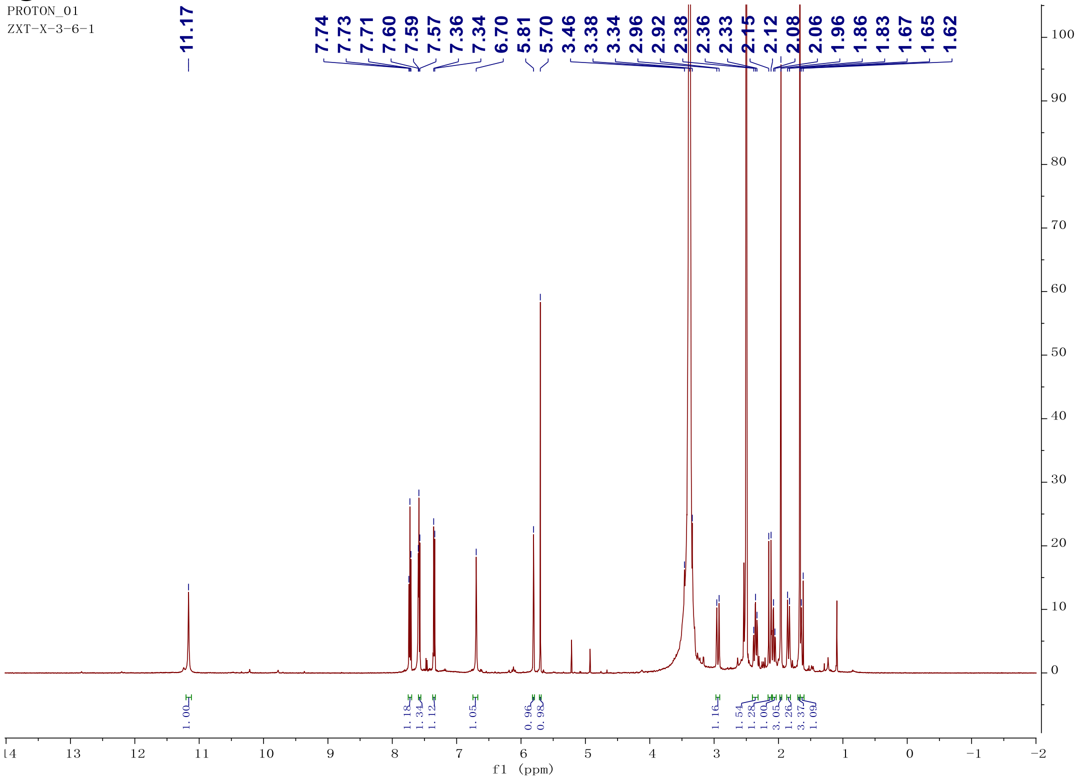

Figure S12. ^{13}C NMR of **2** in DMSO-d_6

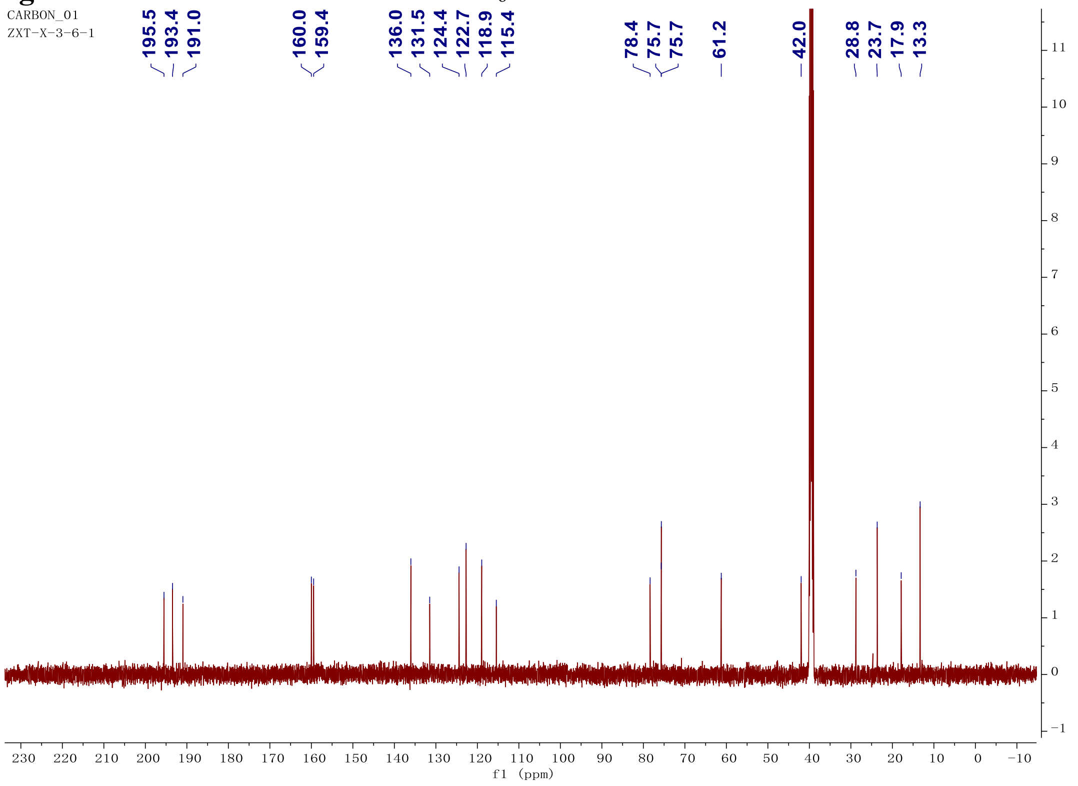

Figure S13. HSQC of **2** in DMSO-d_6

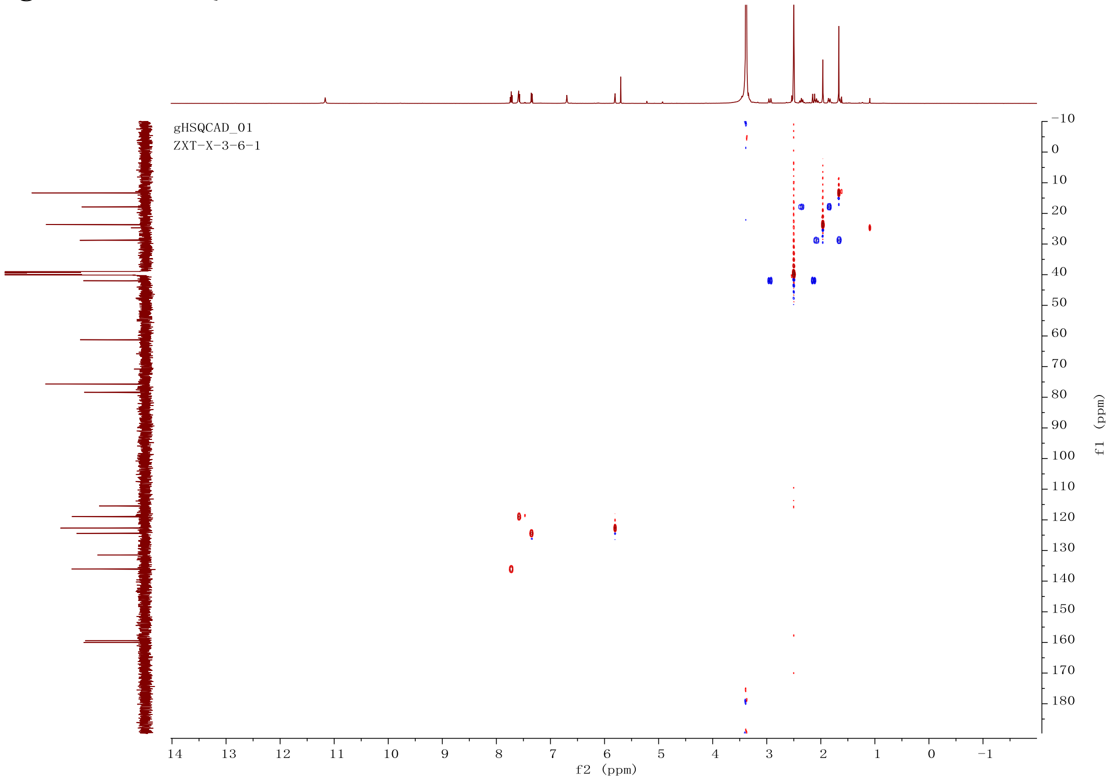

Figure S14. HMBC of **2** in DMSO-d_6

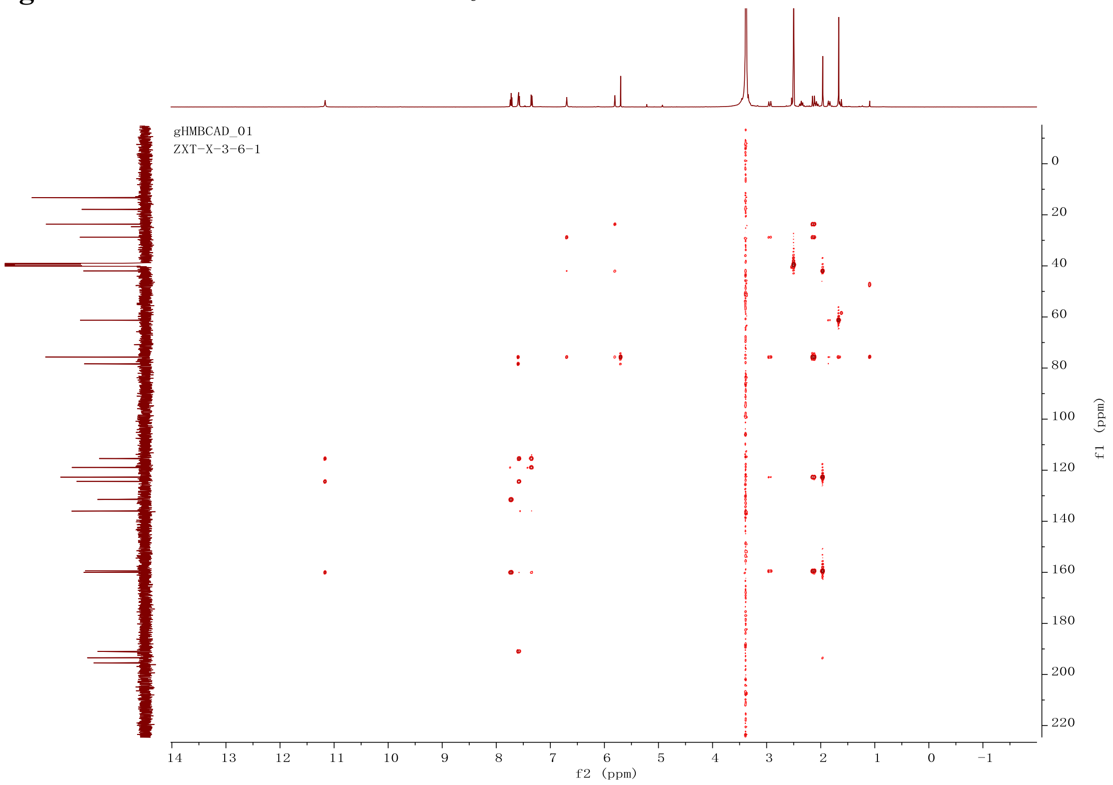

Figure S15. ^{1}H-^{1}H COSY of **2** in DMSO-d_6

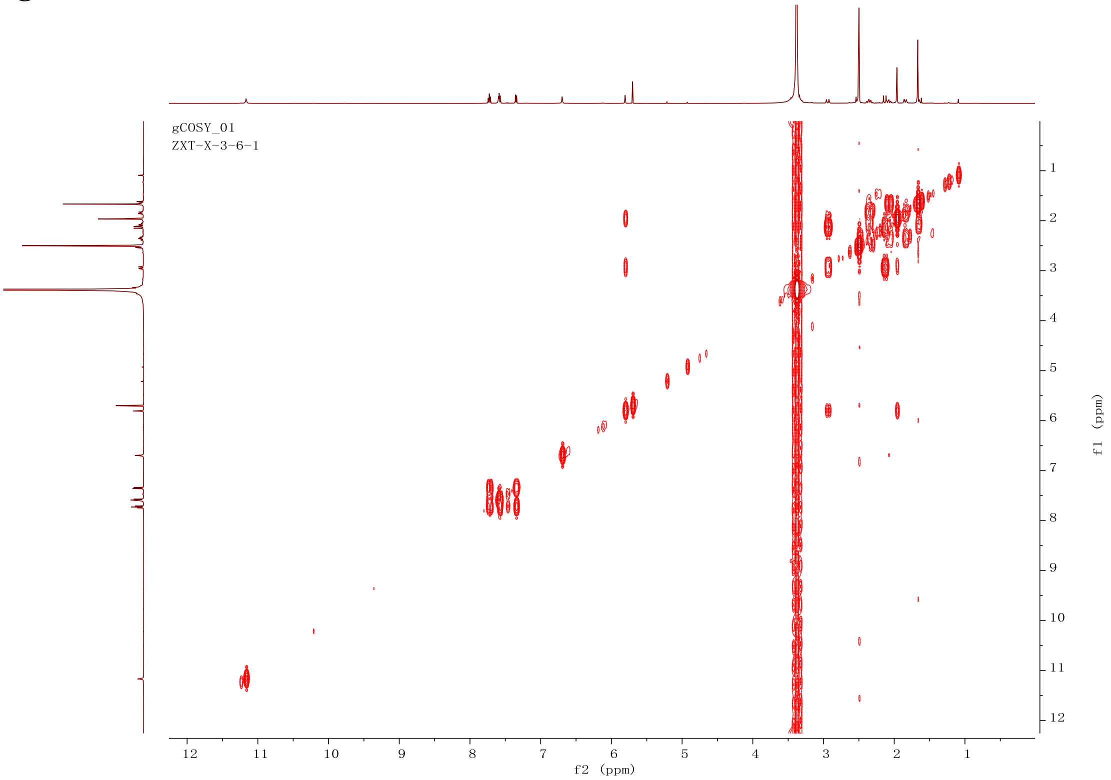

Figure S16. HRESIMS of **2**

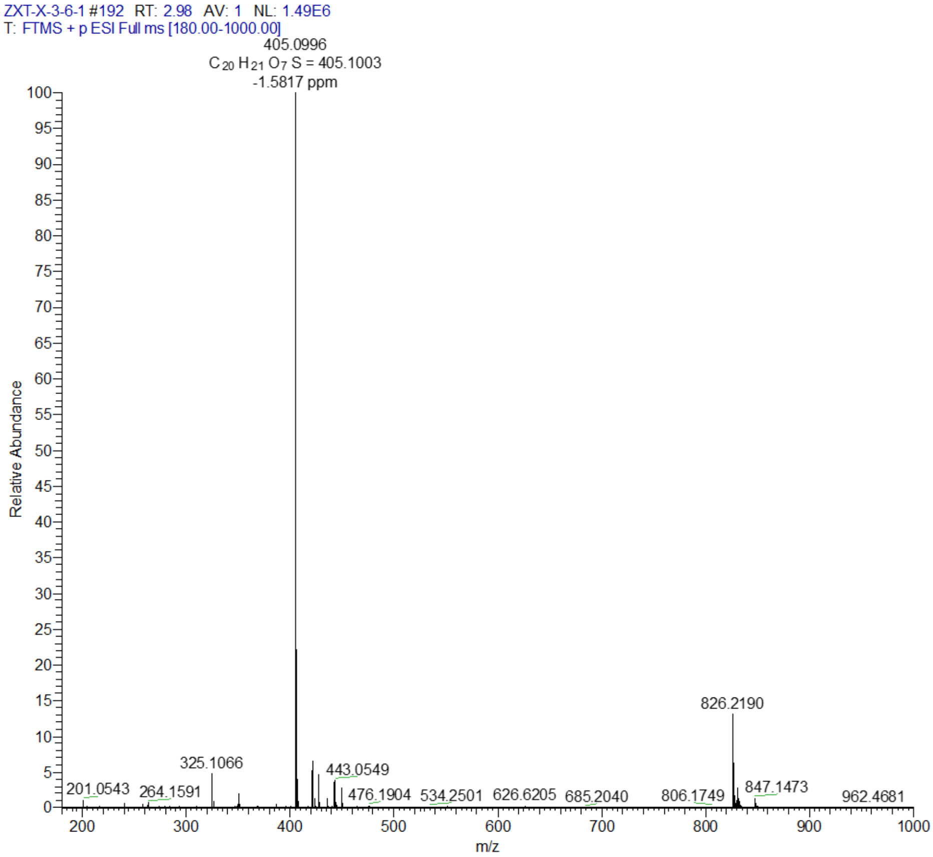

Section S5. NMR and HRESIMS spectra of 3

Figure S17. ^{1}H NMR of **3** in DMSO-d_6

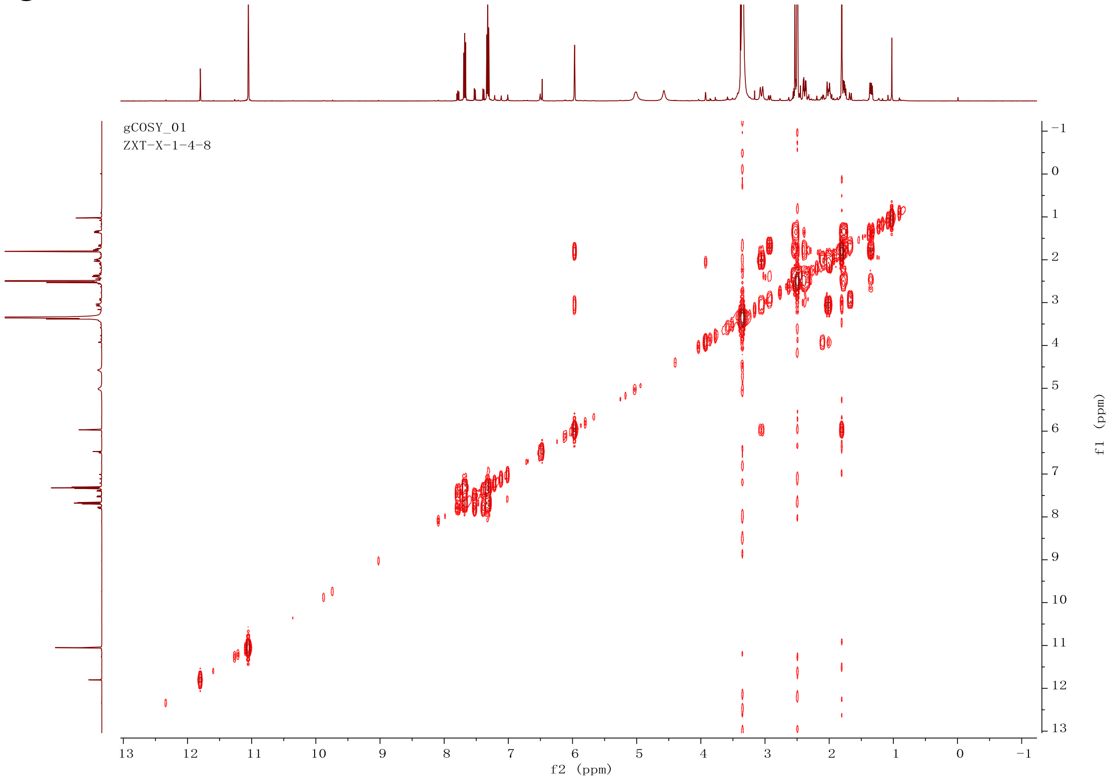

Figure S18. ^{13}C NMR of **3** in DMSO-d_6

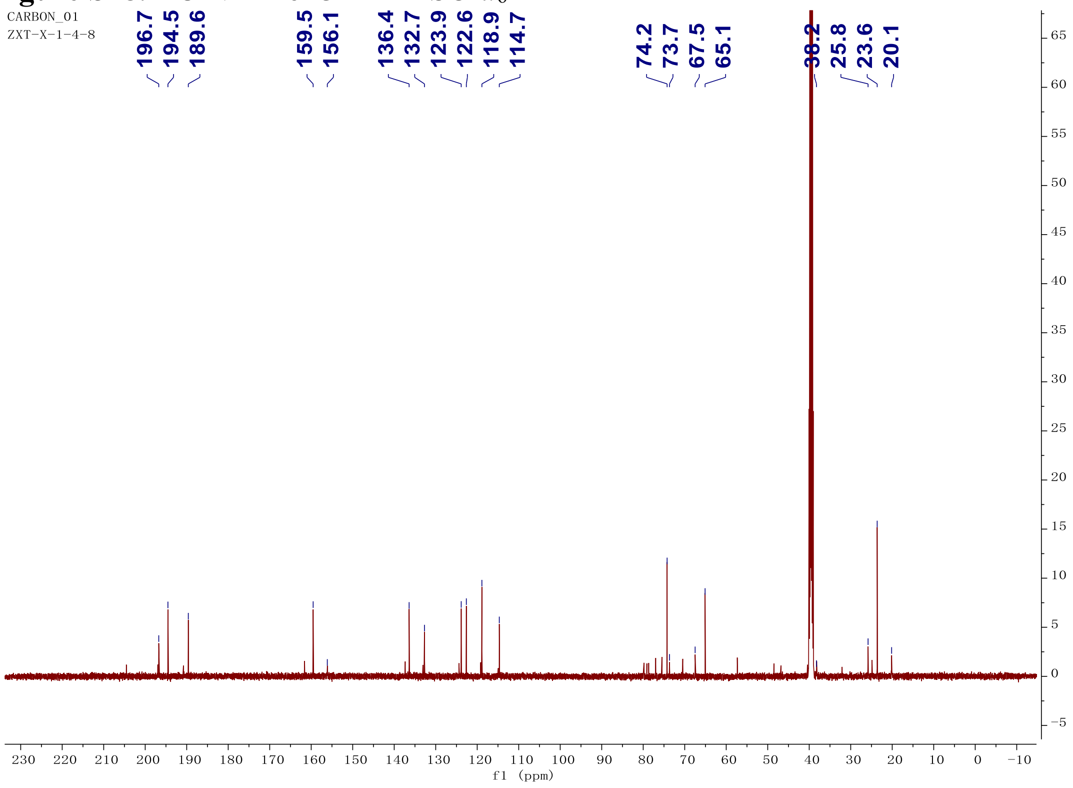

Figure S19. HSQC of **3** in DMSO-*d*6

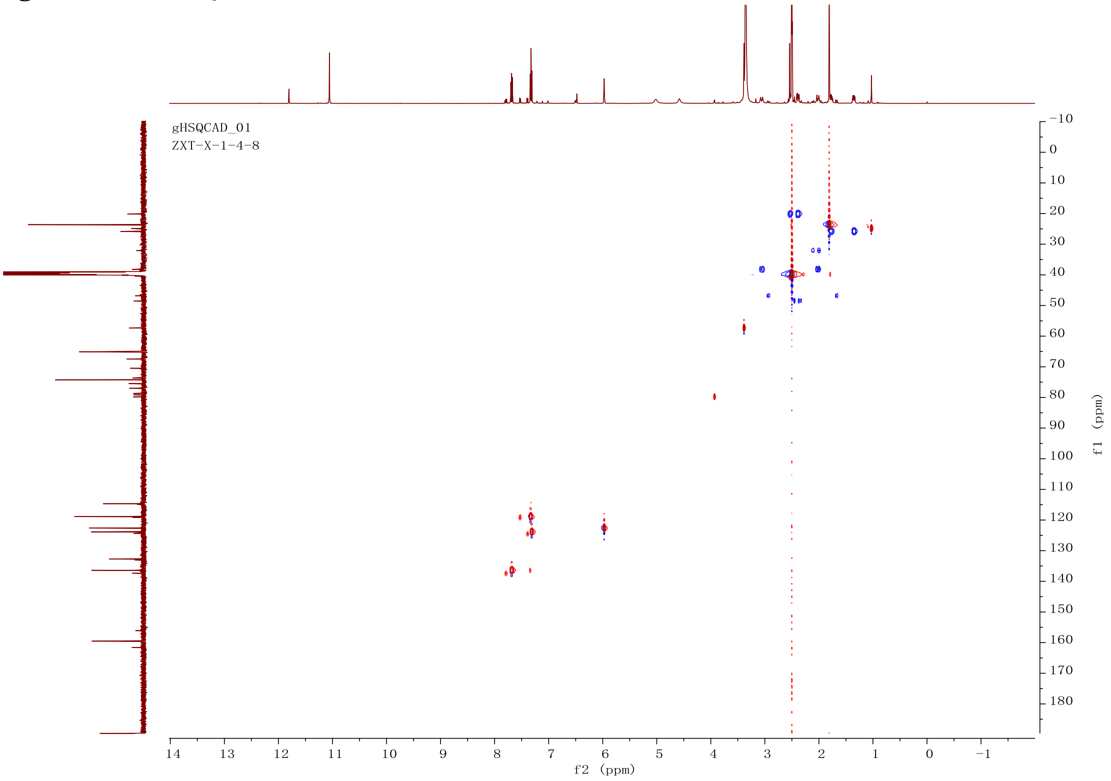

Figure S20. HMBC of **3** in DMSO-*d*6

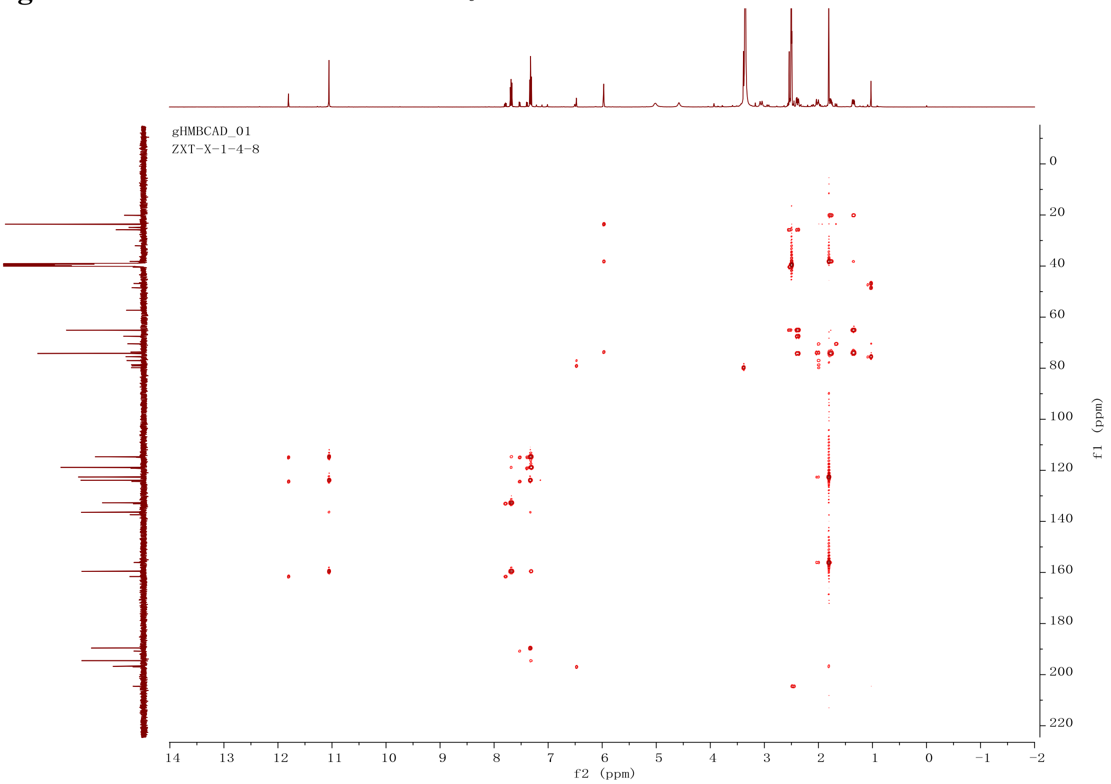

Figure S21. ^{1}H-^{1}H COSY of **3** in DMSO-d_6

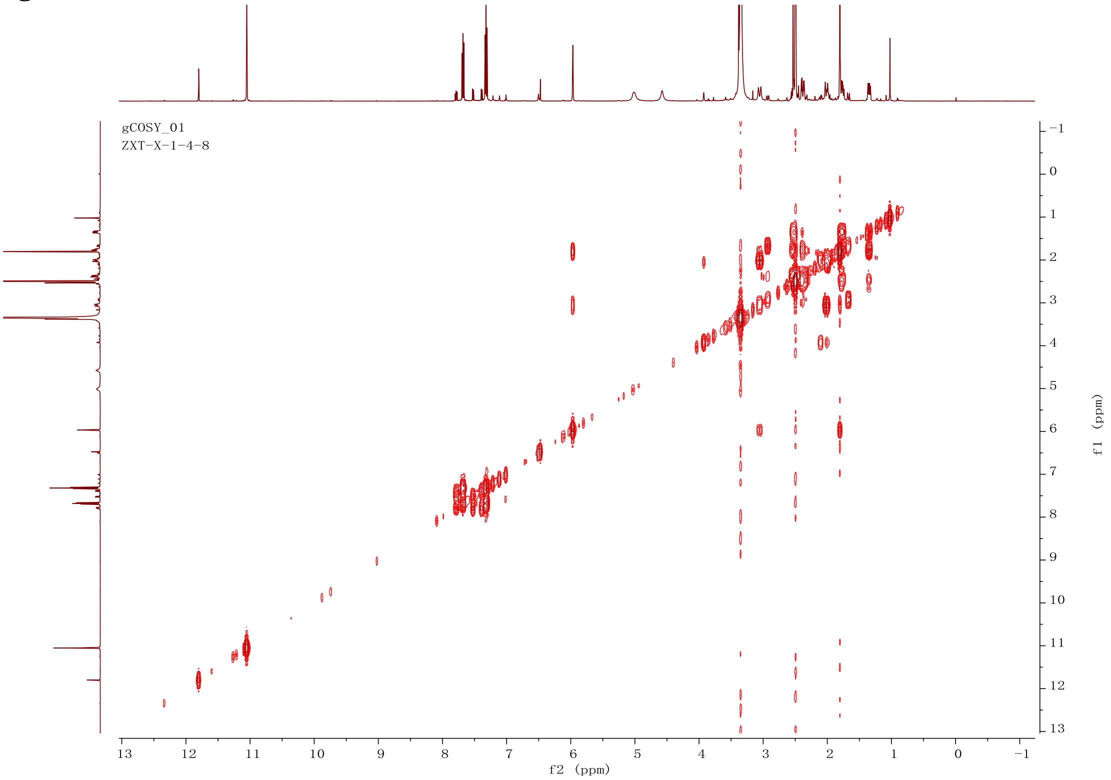

Figure S22. HRESIMS of **3**

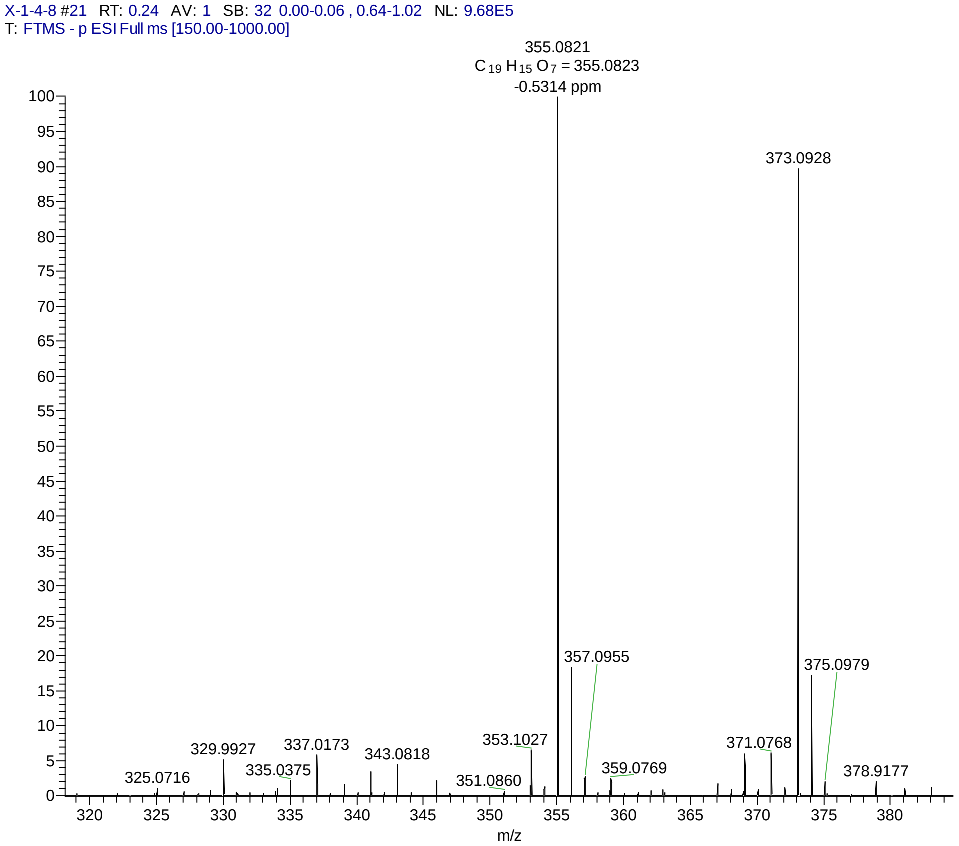

Section S6. NMR and HRESIMS spectra of 4

Figure S23. ^{1}H NMR of **4** in DMSO-d_6

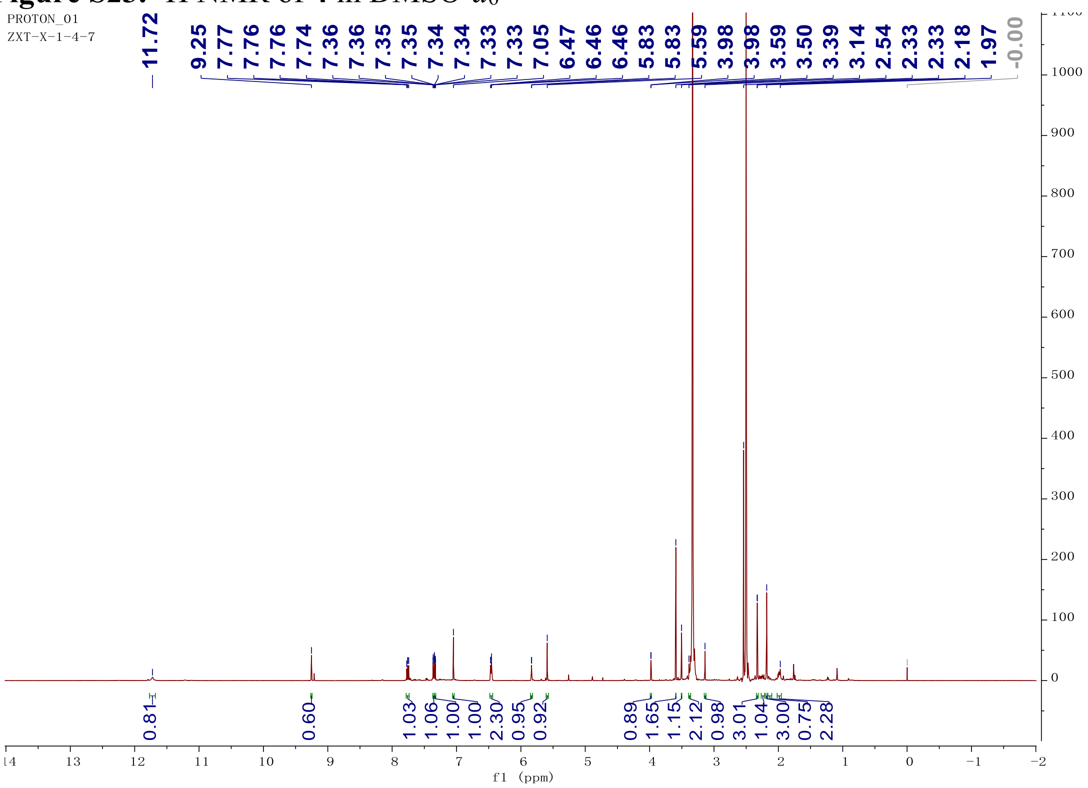

Figure S24. ^{13}C NMR of **4** in DMSO-d_6

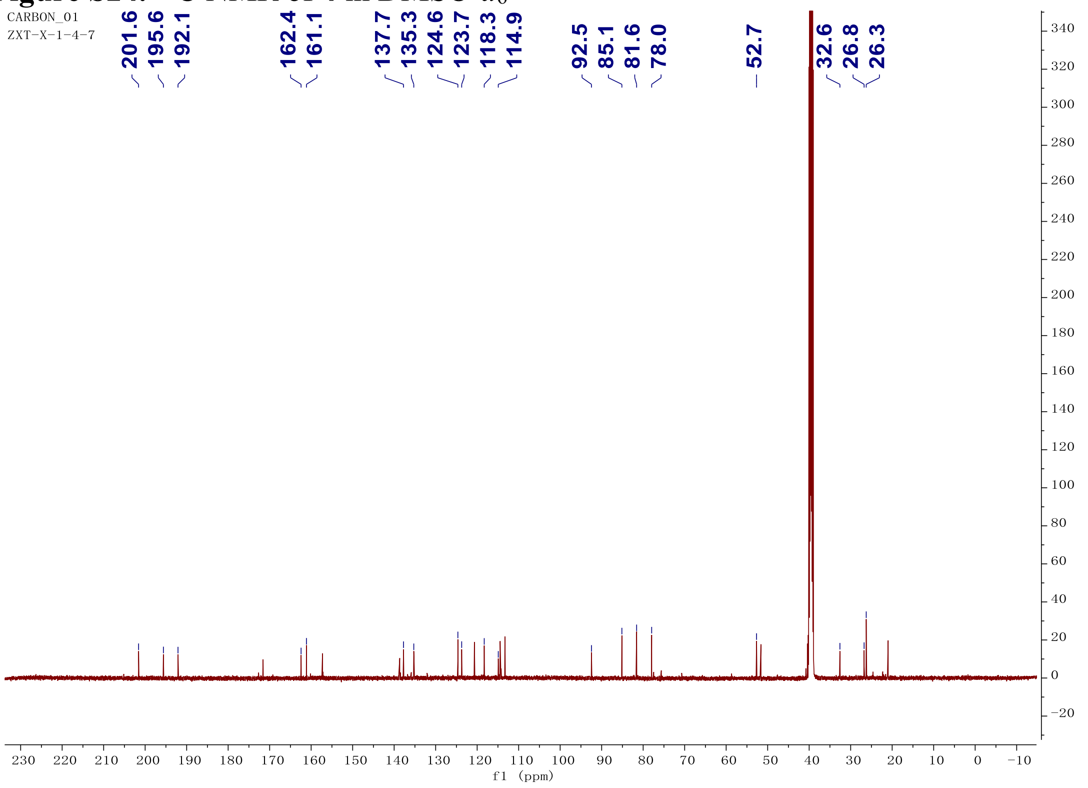

Figure S25. HSQC of **4** in DMSO-d_6

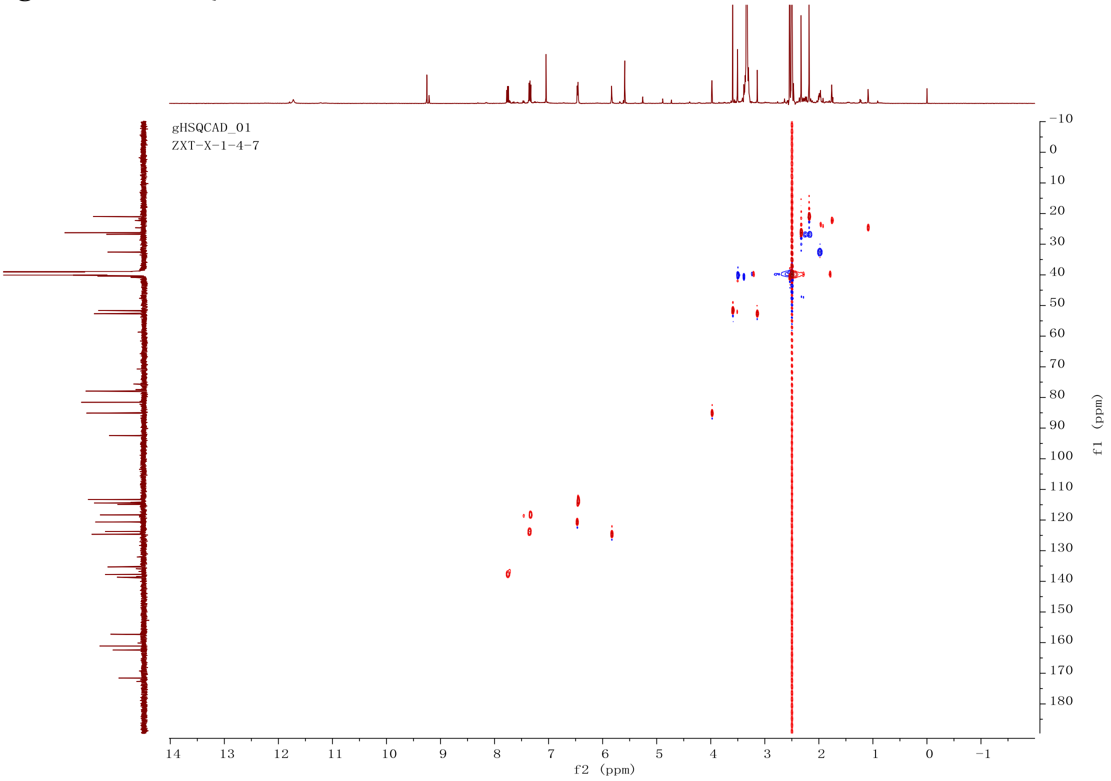

Figure S26. HMBC of **4** in DMSO-d_6

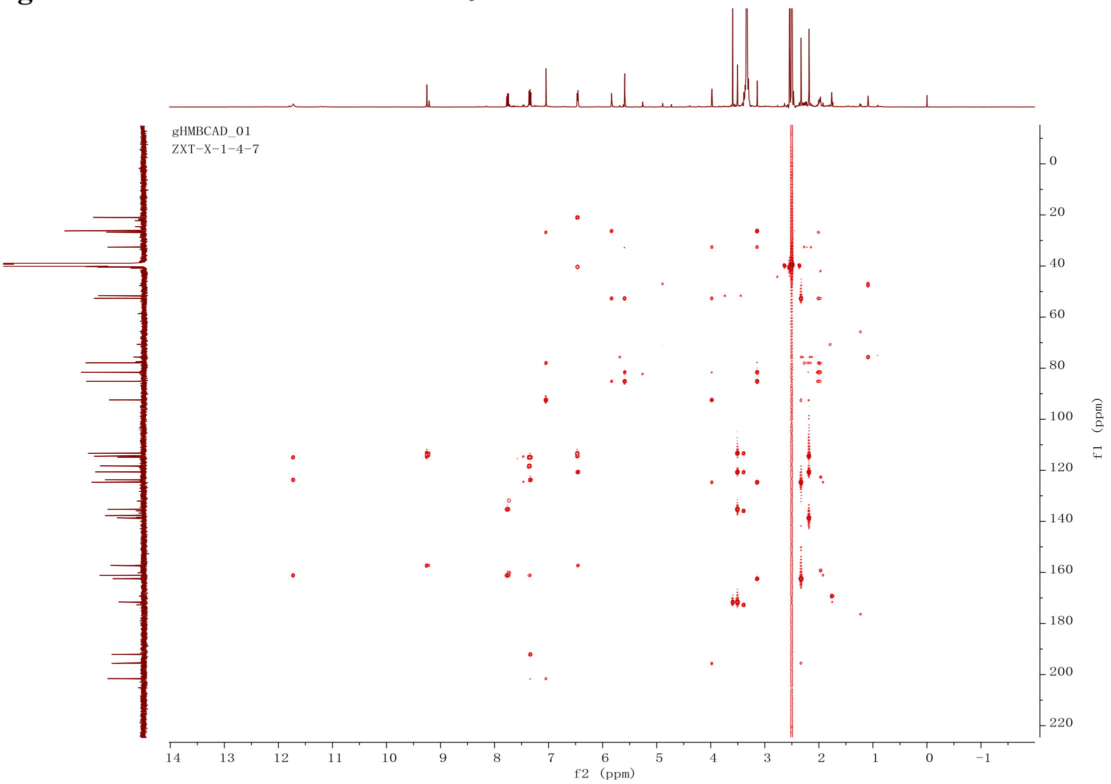

Figure S27. ^{1}H-^{1}H COSY of **4** in DMSO-d_6

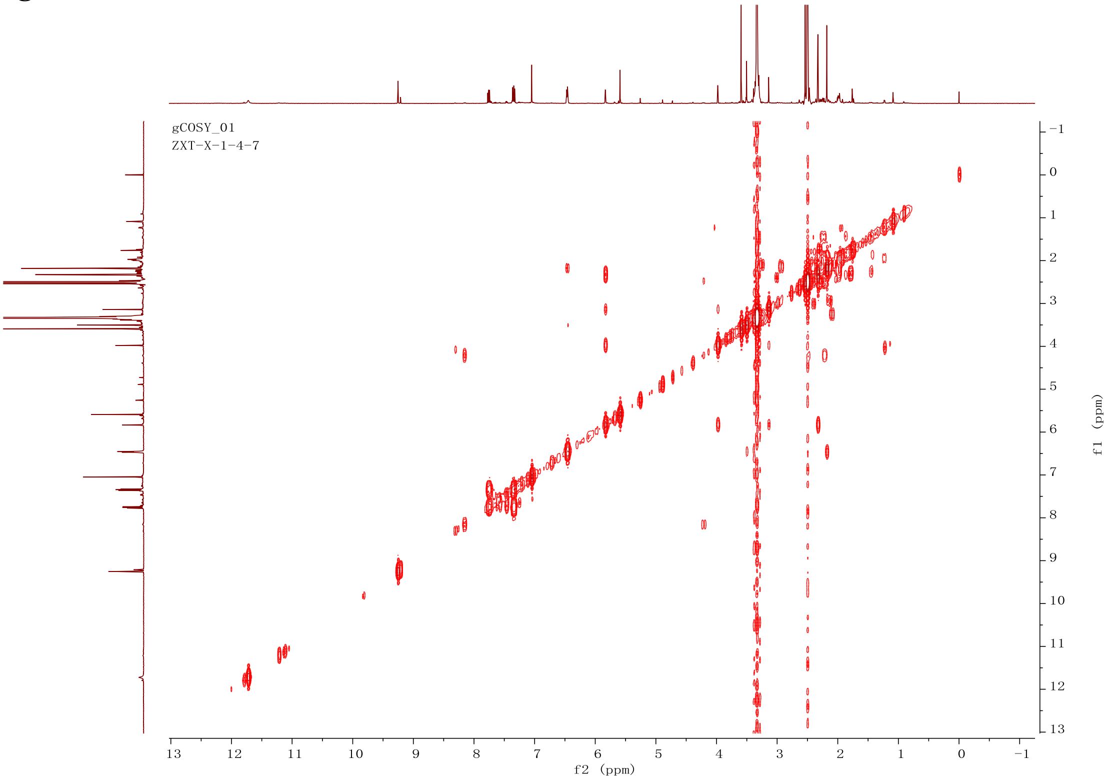

Figure S28. NOESY of **4** in DMSO-d_6

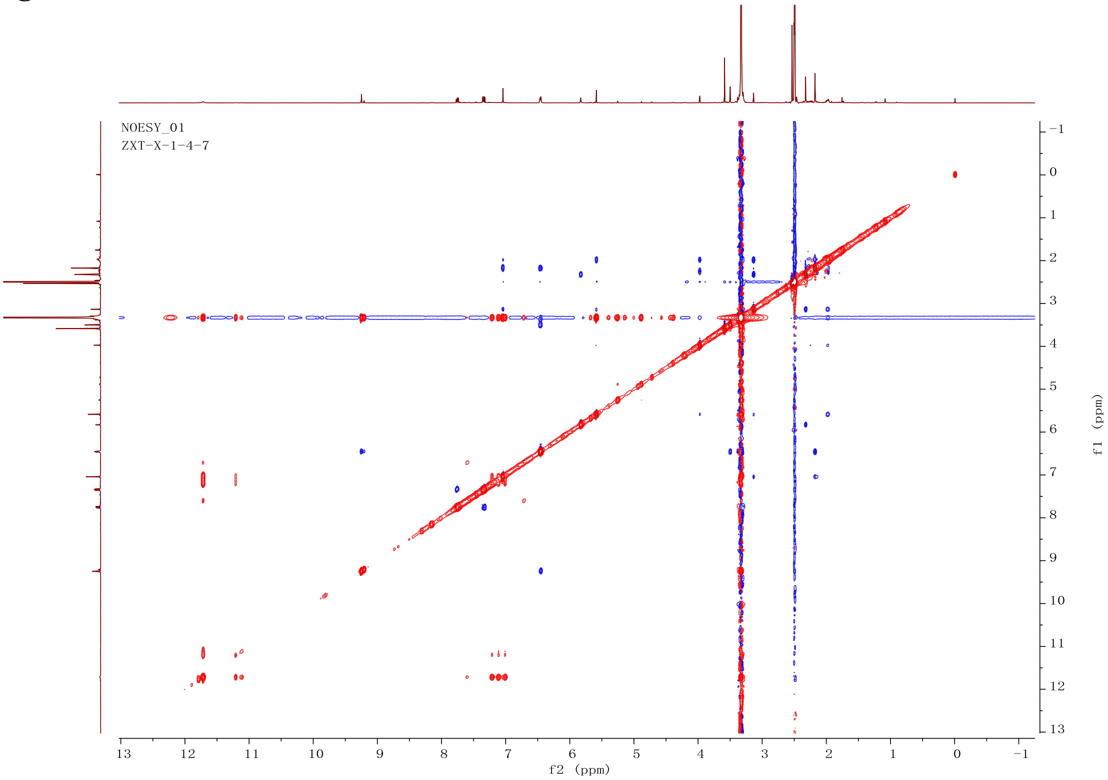

Figure S29. HRESIMS of **4**

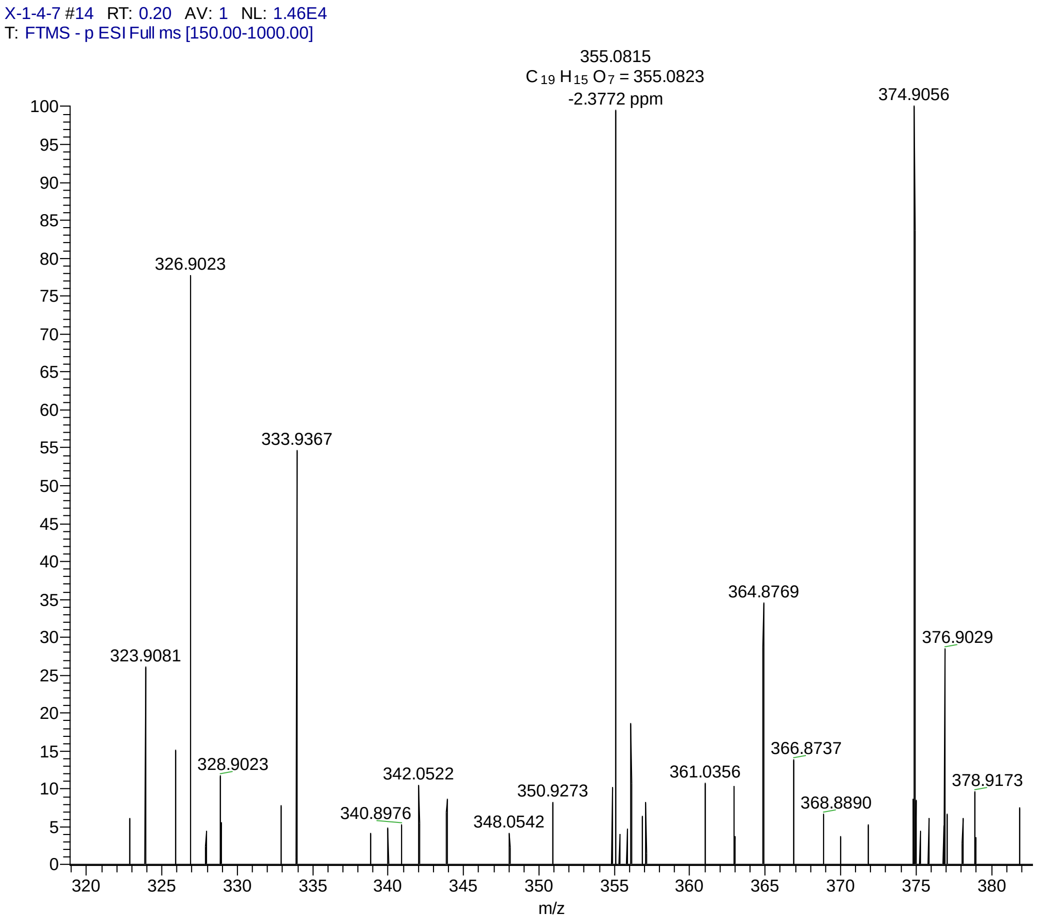

Supplement: Supplementary file 1 [file marinedrugs-22-00480-s001.zip › marinedrugs-3256701-supplementary.pdf]
